# Why Does Child Mortality Decrease With Age? Modeling the Age-Associated Decrease in Mortality Rate Using WHO Metadata From 14 European Countries

**DOI:** 10.3389/fped.2020.527811

**Published:** 2020-10-27

**Authors:** Josef Dolejs, Helena Homolková

**Affiliations:** ^1^Department of Informatics and Quantitative Methods, University of Hradec Králové, Hradec Králové, Czechia; ^2^Division of Pediatric Neurosurgery, Department of Pediatric and Trauma Surgery, Thomayer's Teaching Hospital and Third Faculty of Medicine, Charles University in Prague, Prague, Czechia

**Keywords:** mortality rate, age, childhood, congenital anomalies, WHO database

## Abstract

**Background:** Mortality rate rapidly decreases with age after birth, and, simultaneously, the spectrum of death causes show remarkable changes with age. This study analyzed age-associated decreases in mortality rate from diseases of all main chapters of the 10th revision of the International Classification of Diseases.

**Methods:** The number of deaths was extracted from the mortality database of the World Health Organization. As zero cases could be ascertained for a specific age category, the Halley method was used to calculate the mortality rates in all possible calendar years and in all countries combined.

**Results:** All causes mortality from the 1st day of life to the age of 10 years can be represented by an inverse proportion model with a single parameter. High coefficients of determination were observed for total mortality in all populations (arithmetic mean = 0.9942 and standard deviation = 0.0039).

Slower or no mortality decrease with age was detected in the 1st year of life, while the inverse proportion method was valid for the age range [1, 10) years in most of all main chapters with three exceptions.

- The decrease was faster for the chapter “Certain conditions originating in the perinatal period” (XVI).
- The inverse proportion was valid already from the 1st day for the chapter “Congenital malformations, deformations and chromosomal abnormalities” (XVII).
- The shape of the mortality decrease was very different for the chapter “Neoplasms” (II) and the rates of mortality from neoplasms were age-independent in the age range [1, 10) years in all populations.

**Conclusion:** The theory of congenital individual risks of death is presented and can explain the results. If it is valid, latent congenital impairments may be present among all cases of death that are not related to congenital impairments. All results are based on published data, and the data are presented as a supplement.

## Introduction

Mortality rate is one of the most important indicators of population health. It decreases with age after the birth and increases with age in adults. This increase in adults is approximately exponential and is a curious phenomenon observed in several higher organisms, including *Homo sapiens* (1–9). It is usually interpreted as a manifestation of aging and affects all individuals (10–18). From the statistic point of view, age is a deterministic variable in this relationship and coefficients of determination may be higher than 0.99 (4, 18–23).

**Figure 1:**
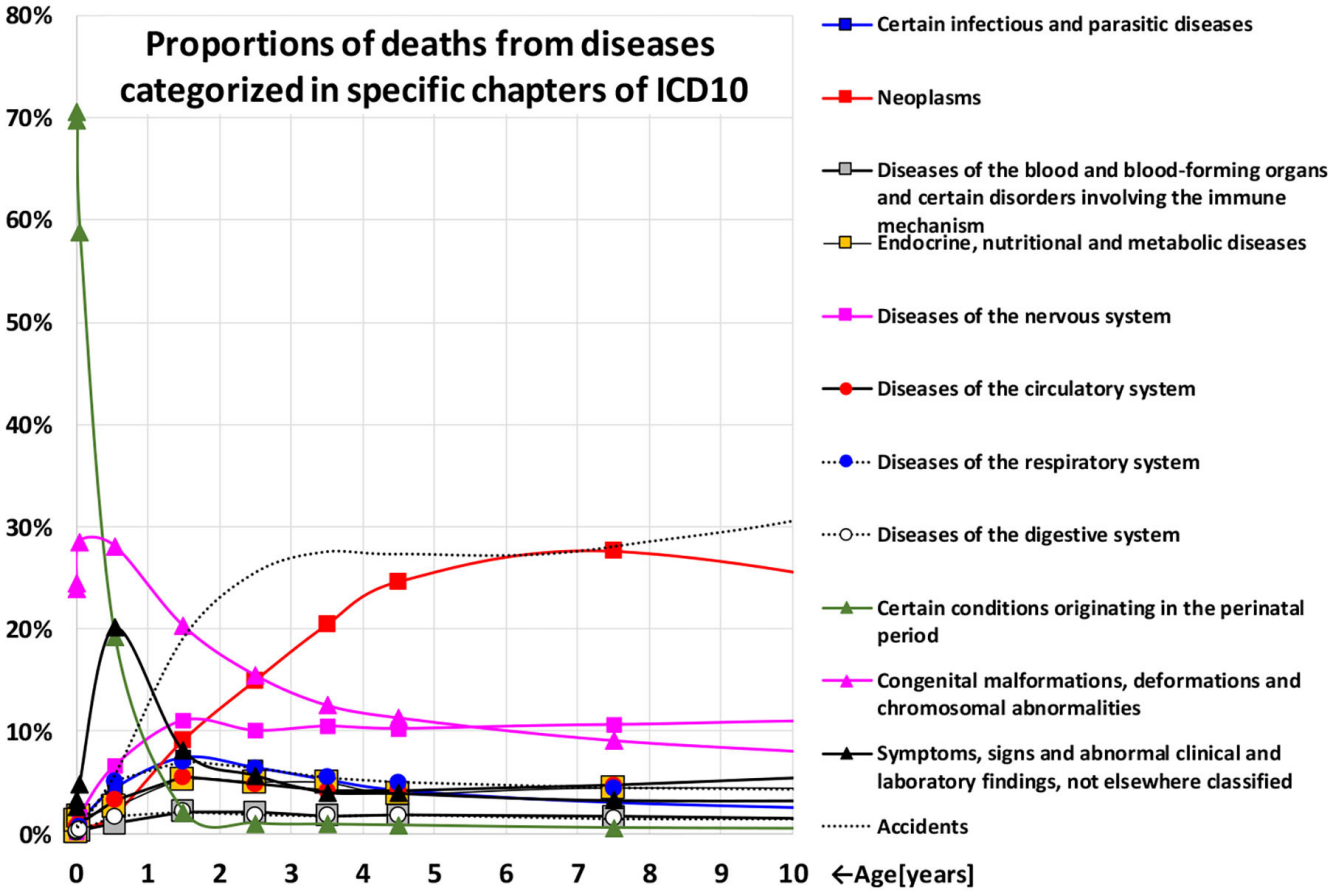
Proportions of deaths from diseases categorized in specific chapters of ICD10.

Faster changes in mortality rates with age are during childhood (5, 7–9). The fast mortality decrease in childhood is accompanied by age-based changes in the spectrum of death causes (24–28). The dominant death causes which reached more than 10% during the 1st weeks of life are insignificant after the age of 5 years. Figure 1 illustrates the proportions of deaths from diseases categorized in specific chapters of the 10th revision of the International Classification of Diseases (ICD10) (28, 29). The proportions are shown in the age categories during the age interval [0, 10) years and 100% corresponds to all deaths in a specific age range. Chapters whose diseases were responsible for more than 1% of the deaths in at least one age category are shown. The proportions were calculated for all 14 countries together and they were very similar in individual countries.

The aims of the study may be split into two main levels:

- What type of model could describe the decrease of mortality with age after birth? Is it exponential, power or some other type of mathematical function? The questions lead to the parametric description of age trajectory of mortality (ATM). The historical list of studies on this topic is not big and the models are described more detailed in the Appendix (the relationships 17–24). The exponential Gompertz model used by Thiele was the first historical attempt (30). Bourgeois Pichat composed the model describing the decrease of death in the age of *n* months for the age interval 28–365 days (31–34). A model of ATM for all age categories was designed later by Heligman and Pollard (7). The simple linear model with two parameters in the log-log scale could be also used and it corresponds to the Weibull distribution [the relationships (23) and (24) in the Appendix]. For example, Bebbington et al. used the reduced additive and flexible Weibull distributions with the separation of mortality to exogenous or endogenous causes over the entire life span (8, 9). It has also been shown that the decrease may be represented by an inverse proportion model with a single parameter, and the model may be valid for higher ages (it has been assessed for congenital anomalies of the central nervous system within the age range [0, 90) years) (28). All-causes ATM and ATM for the main chapters of ICD10 were constructed here and convenient parametric models were found.
- The attempt to explain the steep decline in mortality rate with age after birth represented the second level of the study. The first view could lead to the assumption, that population is homogenous and the development of every individual caused the steep decrease of mortality with age. The alternative assumption that population is very heterogeneous was used here. Heterogeneity from the point of view of ATM was used also in the “frailty model of mortality” in some demographic studies (35, 36). The more theoretical discussion is done for example in Vaupel et al. (36). Vaupel et al. proposed a model of heterogeneity in individual frailty, estimating the gamma distribution of frailty in a population at birth (35). An individual with a frailty of 1 represents a “standard” individual, while an individual with a frailty of 2 is twice as likely to die at any particular age as the standard individual (an individual with a frailty of 0.5, on the other hand, is only one-half as likely to die). It has to be stressed that the theoretical shape of ATM was common to all subpopulations in the theory. Heterogeneity consisted in differences in one of the two parameters of the exponential increase with age and the parameter corresponded frailty. The hypothesis was created to explain the exponential increase of ATM in adults. It was not related to the decrease of mortality with age after birth and the extinction of subpopulations with age was not considered. On the other hand, we have tried to explain the decrease of child mortality with age. We hypothesized that the mortality decrease with age was the result of the extinction of more sever patients. Individuals with more severe congenital impairment typically died during the 1st hours or days of life, and the majority of these severe cases were classified as congenital anomalies or other impairments originating in the perinatal period (28). The proportion of severe congenital anomalies and impairments originating in the perinatal period was very small after the 1st year and decreases to zero as age increases (26). The coincidence between All-causes ATM and ATM constructed for congenital anomalies was the other empirical finding which was assumed here (28). It means that the model of inverse proportion was valid for All-causes ATM, and simultaneously for ATM constructed for congenital anomalies up to 90 years. Furthermore, investigating ATM constructed for other specific group of diseases may help to elucidate how the resulting All-causes ATM was composed. The similarity between ATM constructed for congenital anomalies and other ATM may show that “the sequential exclusion of individuals with more severe impairments was responsible for the steep decrease in total mortality with age.”

From methodical point of view, age was the main explanatory variable here. The age at which an individual died was a very reliable information and it could be assumed that the number of deaths is a well-known information in developed countries. On the other hand, the determination of the death cause may be related to some uncertainty.

The present study used data of 14 European countries and the calendar periods in which ICD10 was used. The calendar periods are shown in Table 1 and the studied population represents ~570 million living persons in one calendar year (the combined population of all the 14 countries is labeled as P14). The total mortality rate decreased three orders of magnitude with age within the age range of [0, 10) years. For example, the highest mortality rate was 39,543.8 per 100,000 living persons per year in P14, in the age range 0–24 h, and the ATM reached the minimum value of 10.5 per 100,000 living persons per year within the age range of [5, 10) years. Mortality rates increased beyond the minimum value, reaching 19,788.2 deaths per 100,000 living persons per year within the age range [90, 95) years (similar figures were seen in each country and in each calendar year).

**Table 1:** Calendar periods and population sizes.

**Country**	**France**	**Germany**	**Italy**	**Spain**	**UK**	**Czech Republic**	**Austria**
Years	2000–2014	1998–2015	2003–2014	1999–2015	2001–2015	1994–2015	2002–2016
Population	61,466,098	80,892,654	59,026,383	44,137,863	61,569,167	10,384,837	8,420,447
**Country**	**Hungary**	**Poland**	**Slovakia**	**Sweden**	**Norway**	**Denmark**	**Finland**
Years	1996–2015	1999–2015	1996–2014	1997–2015	1996–2015	1994–2009	1996–2015
Population	10,055,552	38,570,112	5,407,663	9,215,809	4,708,433	5,357,073	5,265,968

*Size of specific population is mean of population sizes in specific calendar years*.

P14 represents the biggest aggregate for which ATM were constructed and enables smoother curves. P14 may also eliminate zero cases for some diseases in specific age category. Initially, the decrease in ATM was analyzed in the largest population P14. Total mortality rates and mortality rates from diseases of specific ICD10 chapters were used to construct ATM. ATM were also constructed for each country, three specific country groups, and for ICD10 chapters.

## Materials and Methods

The 1-year period is a typical time unit used in epidemiology, biology, and social sciences. Biological events (e.g., death or disease) in different ages may be studied in one specific calendar year across age categories (transversal description). Other possibility is the longitudinal observation of individuals born in the same year (generation study or longitudinal study). If the time unit “1 year” is replaced with the time unit “1 day” (e.g., for some biological, social, or cultural reasons), events observed for 1 year represent the aggregation of the events that occurred in 365 days. Aggregation of more calendar years has a similar meaning. Age was here assumed as the main factor and all other factors were assumed to be less significant. Furthermore, the existence of general mechanisms was assumed, as demonstrated in the ATM after birth. The mechanism could be unknown and was identified as aging in higher age ranges, as mortality rate increased with age (37). For these reasons, ATM were primarily constructed in as large population as possible (P14). Data and results of specific countries and of three country groups were also studied. Including more regions and calendar years within the analysis may eliminate all factors other than age, rendering the impact of age more visible. The following standard definition of the force of mortality at age x was used:

(1) $$\begin{array}{l} \mu \left(x\right)=\underset{h\to 0}{\lim}\frac{D\left(x+h\right)}{L\left(x\right)}={-}^{\frac{dS\left(x\right)}{dx}}{/}_{S\left(x\right)}≅\frac{Di}{Li}.\frac{1}{\left(Bi-Ai\right)} \end{array}$$

where *D*(*x*+*h*) is the number of deaths in a small age range, [x, x+h), the infinitesimal increment *h* is positive. *S*(*x*) is the survival function (percentage of living people at age *x*), which is valid in principle: *S*(*x*) = 1-*F*(*x*), where *F*(*x*) is the cumulative distribution function of the probability of death. The empirical value *Di* is the number of deaths within an age range [*Ai, Bi*), while *Li* is the size of the population among which the deaths occurred. Empirically, changes in *Li* within an age interval [*Ai, Bi*) were very small, when compared with changes in *Di*, and the number of living people *Li* within an age interval [*Ai, Bi*) was used instead of the average number of living people in the region and calendar period. Namely, population *Li* goes through the “window” in time or through the age interval [*Ai, Bi*) and it is the meaning of the product *Li*•(*Bi*-*Ai*) in Equation (1). The unit corresponding to the mortality rate is “person-years,” which means the number of years lived by members of the population between ages *Ai* and *Bi*. Uncertainty or possible demographic error of Li were discussed in the previous studies in detail (20–23) and they were assumed to be negligible with the respect to resulting ATM.

ATM were assumed to be unknown theoretical curves, and were constructed using the right side of the Equation (1). Mortality rates in different age groups describes the groups Li in the same way a decay constant describe the force of a radioactive decay on different radionuclides, while the different radionuclides correspond to the groups Li.

### Population of 14 Countries (P14)

Zero deaths in a given age group may be the major problem for the construction of ATM from a disease or from a set of diseases grouped in some ICD10 chapters. While zero deaths due to some disease may occur within a specific age category in a specific calendar year, at least one death may occur within the same age category in other calendar year. Consequently, the inclusion of additional calendar years may remove this obstacle. The method was first utilized by the well-known astronomer and mathematician Edmond Halley (38–40). In 1693, he elaborated a population life table for the city of Breslau using data based on his analysis of the number of births and deaths recorded in parish registers over several calendar years. This method enables the calculation of the mortality rate within one age category based on the number of deaths and living persons in several years (26, 28, 39, 40). It simply means that, in each age interval, the number of living people and the number of all died in more than one calendar year and more than one region are added together. It is noteworthy that standard epidemiologic and demographic interpretations of the specific value of mortality rate within a specific age category differ from the values calculated for one calendar year. However, the interpretation is the same for all age categories, and the resulting ATM may be interpreted as a hallmark of the population changes with aging. It has also been used in paleodemography, where a population and its corresponding life table were constructed (40). Besides, including more regions and calendar years may eliminate factors other than age.

The WHO mortality database contains the number of deaths within specific age categories in different countries (41). It is crucial that the database uses the following four age categories for the 1st year of life: [0, 24) hours, [1, 7) days, [7, 28) days, and [28, 365) days. Unfortunately, these four categories are not used in all countries and calendar years. The death cause is determined using the specific revision of the International Classification of Diseases in the database. The database contains the 7–10th revisions of ICD and specific revisions are used for different calendar years by different countries. Here, the calendar period used in each country corresponds to the period when the recent 10th revision (ICD10) was applied (29).

The present study used WHO data collected in 14 European countries. Calendar periods and these populations are shown in Table 1 (42). The population of the 14 European countries combined (P14) represents ~570 million living persons in one calendar year. Besides being constructed for populations of specific countries and for P14, ATM were also constructed for the following populations: (a) the five big EU countries (France, Germany, Italy, Spain and the U.K.), labeled as “P1,” (b) the five Central European countries (Austria, Czech Republic, Hungary, Poland and Slovakia), labeled as “P2,” and (c) the four Nordic countries (Denmark, Finland, Norway and Sweden), labeled as “P3.” The relevance order was: P14, the three subgroups of countries (P1, P2, and P3), and the 14 countries separately. A total of 18 ATM could be constructed for specific groups of diseases in 18 different populations. If no deaths were registered within a specific age category in any population, ATM for this population was not constructed. For example, no cases in the chapter “Pregnancy, childbirth, and the puerperium” (XV) were found within the age range [0, 10) years in all populations. Thus, this chapter was not relevant to the study.

As the WHO database (41) is not user-friendly, “txt” files were imported into Microsoft (MS) Excel 2016. Moreover, a simple program was developed using Visual Basic for Applications to perform the following manipulations (the calculation of the sum of cases in each chapter and each age category). MS Excel was used at the first level of processing, along with standard packages in R 3.3.2 for Windows. The level of statistical significance was set to 0.05 for all tests. The arithmetic mean of the interval endpoints was used as a representative value for each age category. The time unit “1 year” was used in all age categories and in all calculations.

### Model Assessment

The decrease in the mortality rate with age was visually linear in the log–log scale. At first, the linearity of the trajectory within the age range [0, 10) years was statistically examined by the full quadratic model (2) using the method of least squares (LS):

(2) $$\begin{array}{l} ln\left[\mu \left(x\right)\right]=constant+\gamma .\ln\;\left(x\right)+\delta .\ln\;\left(x\right).\text{ln}\left(x\right) \end{array}$$

The null hypothesis for the quadratic element (H_o_: γ = 0) was not rejected (*P* > 0.05), while the parameter γ was significant (*P* < 0.0001) in all populations. The F-test, in which the full model (2) does not provide a significantly better fit than the restricted model without the quadratic element, provided the same result. Consequently, it was assumed that the decrease in total mortality with age was linear in the log–log scale. Following this assumption, a linear submodel (3) was developed:

(3) $$\begin{array}{l} ln\left[\mu \left(x\right)\right]=constant+\gamma .\ln\;\left(x\right) \end{array}$$

The two parameters ln[μ_1_], γ, their standard deviations, and the adjusted coefficients of determination *R*^2^ in the linear model (3) were calculated using the LS method for the age interval of [0, 10) years. The hypothesis that the residuals were age-independent was not rejected (*P* > 0.05), and our data further confirmed that the residuals were not *U*-shaped. The results are shown in Table 2.

**Table 2:** Results of total mortality calculated in the log-log scale.

**Population**	**Test of linearity**	**γ**	**Lower CI 95%**	**Upper CI 95%**	**${\bar{R}}^{2}$**	**$R_{\text{b}}^{2}$**	**$R_{\text{b}}^{2}$-${\bar{R}}^{2}$**	**Test of c/x**
France	0.31	−0.984	−1.034	0.934	0.9963	0.9965	0.0002	0.47
Germany	0.70	−0.987	−1.036	0.938	0.9964	0.9967	0.0003	0.54
Italy	0.82	−1.007	−1.072	0.942	0.9941	0.9948	0.0007	0.81
Spain	0.36	−0.960	−1.022	0.898	0.9941	0.9931	−0.0010	0.17
UK	0.88	−1.030	−1.082	0.977	0.9963	0.9959	−0.0004	0.22
**P1**	0.72	−0.997	−1.046	0.948	0.9965	0.9970	0.0004	0.87
Czechia	0.18	−0.918	−1.004	0.831	0.9873	0.9810	−0.0064	0.06
Austria	0.14	−1.006	−1.069	0.944	0.9944	0.9951	0.0007	0.82
Hungary	0.21	−1.008	−1.075	0.941	0.9937	0.9945	0.0007	0.79
Poland	0.93	−1.023	−1.086	0.961	0.9946	0.9948	0.0002	0.41
Slovakia	0.20	−0.951	−1.030	0.871	0.9901	0.9887	−0.0014	0.19
**P2**	0.73	−1.003	−1.063	0.942	0.9948	0.9955	0.0006	0.92
Sweden	0.99	−0.968	−1.023	0.912	0.9953	0.9948	−0.0005	0.22
Norway	0.78	−0.966	−1.012	0.920	0.9967	0.9959	−0.0008	0.13
Denmark	0.29	−1.014	−1.060	0.967	0.9970	0.9972	0.0002	0.51
Finland	0.08	−0.960	−1.032	0.888	0.9919	0.9913	−0.0007	0.23
**P3**	0.45	−0.975	−1.027	0.924	0.9960	0.9959	−0.0001	0.30
**P14**	0.78	−0.996	−1.048	0.945	0.9962	0.9967	0.0005	0.88

*The column labeled as “Test of linearity” contains p-value of the test of the null hypothesis for the quadratic element (H_o_: δ = 0) in model (2) (all trajectories were linear in the log-log scale). The column labeled as “γ” contains point estimation of the slope in model (3) and the next two columns contain the limits of 95% confidence intervals of the parameter γ. ${\bar{R}}^{2}$ is the adjusted coefficient of determination calculated for two parameters and nine points in the model (3). ${\text{R}}_{\text{b}}^{2}$ is the coefficient of determination of the inverse proportion (4) in the log-log scale and it was calculated using formula (6). The last column labeled as “Test of c/x” contains p-value of the standard Fisher's test, which determined that the model (3) (with two parameters) does not provide a significantly better fit than the model (4) (with a single parameter). The inverse proportion (4) was a successful model in all cases*.

Because the slope γ was close to the value−1, the null hypothesis H_o_: γ = −1 was examined using the model (3) and was not rejected (*P* > 0.05). The specific value −1 for the parameter γ corresponds to the inverse proportion between mortality rate and age. If γ = −1, the following is formally valid:

(4) $$\begin{array}{l} \mu \left(x\right)=\frac{\mu_{1}}{x}\text{ is on the log}-\text{log scale}:\text{ln}\left[\mu \left(x\right)\right]\\ \;=\text{ln}\left(\mu_{1}\right)-\text{ln}\left(x\right) \end{array}$$

The parameter μ_1_ in model (4) can be estimated using the Equation (5) for *n* pairs of values ln[μ(*x*_*i*_)] and ln(*x*_*i*_), via the LS method:

(5) $$\begin{array}{l} ln\left(\mu_{1}\right){=}^{\sum \left\{ln\left[\mu \left(x_{i}\right)\right]+ln\left(x_{i}\right)\right\}}{/}_{n} \end{array}$$

Furthermore, the standard coefficient of determination ${\text{R}}_{\text{b}}^{2}$ in model (4) can be described as follows:

(6) $$\begin{array}{l} R_{b}^{2}=1{-}^{SS_{resid}}{/}_{SS_{Total}}=\\ \;=1{-}^{\sum {\left\{ln\left[\mu \left(x_{i}\right)\right]-\left[ln\left(\mu_{1}\right)\;-ln\left(x_{i}\right)\right]\right\}}^{2}}{/}_{\sum {\left\{ln\left[\mu \left(x_{i}\right)\right]{-}^{\sum ln\left[\mu \left(x_{i}\right)\right]}{/}_{n}\right\}}^{2}} \end{array}$$

The inverse proportion (4) is a nested model that includes the two-parameter linear model (3). In the next step, the null hypothesis that model (3) (with two parameters) does not provide a significantly better fit than model (4) (with a single parameter), was tested using a standard Fisher's test. Resulting *p*-values are shown in Table 2 (the null hypothesis was not rejected in any population). Consequently, the inverse proportion between total mortality and age was the resulting model within the age range of [0, 10) years. Besides, the standard coefficients of determination ${\text{R}}_{\text{b}}^{2}$ in model (4) were higher than 0.99 in all populations, except for Czech Republic (0.981). Arithmetic mean of coefficients of determination ${\text{R}}_{\text{b}}^{2}$ in model (4) was 0.9942 and standard deviation was 0.0039. Such values are uncommon and the inverse proportion between total mortality and age may be considered as a deterministic relationship. It implies that, compared to the 1st day of life, mortality rate was 10 times lower after 10 days, 100 times lower after 100 days, and 3,650 times lower after 10 years.

## Results

### Age Trajectory of Total Mortality

Age trajectory of total mortality is more important than ATM due to specific groups of diseases. It is also more reliable because the determination of the cause of death may be burden with some uncertainty (for example, it may be expected for cases registered in the 18th chapter of the ICD10: “Symptoms, signs and abnormal clinical and laboratory findings, not elsewhere classified”). The age trajectories of total mortality are expected to be smoother and show more significant findings than the investigations of specific diseases.

**Figure 2:**
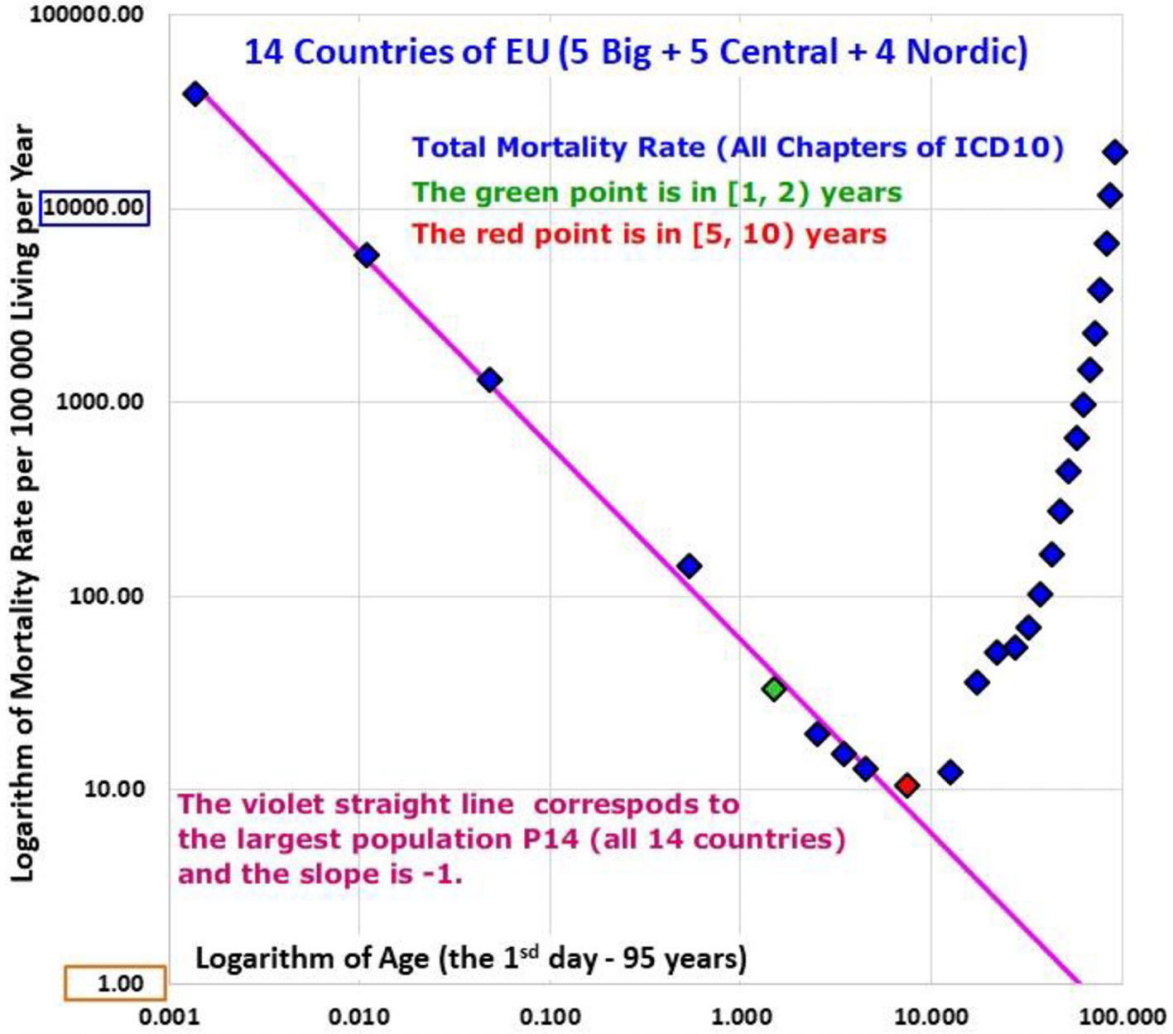
(Animation 1) Age trajectory of total mortality.

The age trajectories of total mortality reached the minimal value within the age range [5, 10) years in each country separately, as well as in P1, P2, P3, and P14. Slovakia was an exception, where the minimal value was found within the age range [10, 15) years. Furthermore, the age range [0, 10) years was used to evaluate the total mortality decrease with age. At first, the age trajectories of total mortality were investigated visually. All age trajectories of total mortality are shown in Animation 1 and one age trajectory of total mortality for P14 is shown in Figure 2 in the log-log scale.

One of *p*-values of the Test of linearity in 14 separate countries was only 0.08 in Finland and one of *p*-values of the Test of c/x was 0.06 in Czechia in Table 2. The relatively small values near the significance level 0.05 showed that the null hypothesis of the linearity was more in danger in Finland. The null hypothesis that the model c/x described data was more in danger in Czechia. On the other hand, *p*-values of both tests were very high in aggregated populations P1, P2, P3, and P14 in Table 2 (it simply corresponded to the law of large numbers and to the fact that populations P1, P2, P3, and P14 were composed from smaller populations). It has to be note that all results and conclusions were limited to the fact that the investigation populations originated from Central, Western and Northern European countries and may not be representative of non-European countries. The partly similar study was done in the six countries of the South America and it had showed that the model of inverse proportion was valid for All causes ATM (specific chapters of ICD10 were not investigated in the study) (23). It indicated that the conclusion of the presented study may be valid in less developed countries. Naturally, other study has to confirm or reject such findings.

### Chapters of the ICD10

Cause of death is a less reliable information than the determination of age. On the other hand, the inspection of ATM from specific diseases may provide an important viewpoint to the decrease of mortality rate with age. It may show the composition of age trajectory of total mortality. The WHO database (41) uses the ICD10 classification (29), and the four age categories during the 1st year of life were used in some calendar years and regions. If the four age categories are used, the ATM may be constructed (constructing ATM with <4 age ranges for the 1st year of life is also possible; however, essential information about decreased mortality rate after birth is lost in such case).

The main chapters of the ICD10 were used here to study the spectrum of diseases related to the decrease in mortality rate with age after birth. Besides, ATM due to congenital anomalies of the central nervous system because higher coefficient of determination in some previous studies [the category is the subset of the chapter “Congenital malformations, deformations and chromosomal abnormalities” (XVII.)].

Table 3 contains the number of deaths within the age interval [0, 10) years and the proportions of diseases of main chapters of the ICD10 calculated for the P14. If non-zero cases were present in each age category, ATM could be constructed, and such chapter was labeled as “yes” in the column “ATM.” All external causes (accidents) were mentioned in the chapters XIX, XX, and XXI, and were grouped together in a single category labeled as “Accidents.” The two dominant chapters “Certain conditions originating in the perinatal period” (XVI) and “Congenital malformations, deformations and chromosomal abnormalities” (XVII) addressed cases of congenital abnormality or any other defect developed during the perinatal period (diseases of the two chapters accounted for 39 and 23% of all deaths in the P14 within the age interval [0, 10) years, respectively). The chapter “Symptoms, signs and abnormal clinical and laboratory findings, not elsewhere classified” (XVIII) addressed cases with unclear diagnosis and was evaluated separately (it may be assumed that visible congenital abnormalities were not related to death in this chapter).

**Table 3:** Results calculated in main chapters of ICD10 during the first 10 years in P14.

**Chapter**	**∑Di**	**P**	**ATM**	**the 1st day-365 days**			**1–10 years**		
				**γ_1_**	**γ_1_ = 0**	**γ_1_ = −1**	**γ_2_**	**γ_2_ = 0**	**γ_2_ = −1**
Certain infectious and parasitic diseases (I.)	9,224	2.49	Yes	−0.22	0.011	0.022	−1.28	0.000	0.006
Neoplasms (II.)	20,754	5.61	Yes	−0.65	0.026	Ho	−0.001	Ho	0.0002
Diseases of the blood and blood–forming organs and certain disorders involving the immune mechanism (III.)	2,871	0.78	Yes	−0.50	0.011	0.011	−0.88	0.003	0.295
Endocrine, nutritional and metabolic diseases (IV.)	8,306	2.25	Yes	−0.45	Ho	Ho	−0.85	0.007	Ho
Mental and behavioral disorders (V.)	215	0.06	No	x		x	x		x
Diseases of the nervous system (VI.)	16,854	4.56	Yes	−0.25	0.020	0.002	−0.73	0.006	Ho
Diseases of the eye and adnexa (VII.)	4	0.00	No	x	x	x	x	x	x
Diseases of the ear and mastoid process (VIII.)	158	0.04	No	x	x	x	x	x	x
Diseases of the circulatory system (IX.)	8,374	2.26	Yes	−0.49	0.006	0.005	−0.83	0.014	Ho
Diseases of the respiratory system (X.)	10,176	2.75	Yes	−0.22	Ho	0.016	−1.01	0.001	Ho
Diseases of the digestive system (XI.)	3,454	0.93	Yes	−0.43	0.012	0.007	−0.92	0.002	Ho
Diseases of the skin and subcutaneous tissue (XII.)	46	0.01	No	x	x	x	x	x	x
Diseases of the musculoskeletal system and connective tissue (XIII.)	331	0.09	No	x	x	x	x	x	x
Diseases of the genitourinary system (XIV.)	758	0.20	Yes	−0.46	0.013	0.009	−0.64	0.005	0.023
Pregnancy, childbirth and the puerperium (XV.)	0	0.00	No	x	x	x	x	x	x
Certain conditions originating in the perinatal period (XVI.)	144,469	39.06	Yes	−1.16	0.005	Ho	−1.52	0.004	Ho
Congenital malformations, deformations and chromosomal abnormalities (XVII.)	85,252	23.05	Yes	−0.91	0.0001	Ho	−1.22	0.001	Ho
Symptoms, signs and abnormal clinical and laboratory findings, not elsewhere classified (XVIII.)	29,132	7.88	Yes	−0.62	0.042	Ho	−1.29	0.003	Ho
Accidents (XIX,.XX.,XXI.)	29,487	7.97	Yes	x	x	x	x	x	x
“Other diseases”	60,771	16.43	Yes	−0.36	0.007	0.002	Ho	0.003	Ho
CACNS (ICD10 codes: Q00–Q07)	10,596	2.86	Yes	−0.99	0.007	Ho	Ho	0.002	Ho

*Diseases from chapters for which the decrease in mortality was slower during the 1st year than after the 1st year and were not related to congenital impairment were added to the group labeled as “Other diseases.” This group contained the chapters I-XV, without the chapter II (neoplasms). The last three chapters (XIX, XX, and XXI) were aggregated into a category labeled as “Accidents.” The column labeled as “∑Di” contains number of deaths within the age range [0, 10) years. The column labeled as “P” contains proportions of diseases of specific chapter or chapters in all deaths within the age interval [0, 10) years. The column labeled as “ATM” indicates if all age categories contained a non-zero number of deaths and if ATM could be constructed for the age range [0, 10) years. If all age categories contained a non-zero number of deaths, the row was marked with “yes” in the column “ATM”. If one age category contained zero deaths, the row was marked with “no” in the column “ATM.” Slope γ_1_ was calculated in the log-log scale for the first four age categories (during the first year). Slope γ_2_ was calculated in the log-log scale for the age range [1, 10) years. The column labeled as “γ_1_ = 0” contains p-value of the test of the null hypothesis Ho: γ_1_ = 0. The column labeled as “γ_1_ = −1” contains p-value of the test of the null hypothesis Ho: γ_1_ = −1. The column labeled as “γ_2_ = 0” contains p-value of the test of the null hypothesis Ho: γ_2_ = 0. The column labeled as “γ_2_ = −1” contains p-value of the test of the null hypothesis Ho: γ_2_ = −1. If the null hypothesis was not rejected, the symbol “Ho” was used in the last four columns (a value of 0.05 indicated statistical significance for all tests)*.

The inverse proportion model (4) fits ATM of total mortality with very high coefficients of determination. Coefficients of determination lower than 0.99 were found only in Czech Republic and Slovakia (arithmetic mean = 0.9942 and standard deviation = 0.0039 and a value of 0.9967 was reached in P14). Simultaneously, total mortality rates were the sum of deaths registered in all chapters of the ICD10. Thus, the whole spectrum of ATM due to the specific chapters shows the composition of the age trajectory of total mortality. For these reasons, preference was given to the linear relationship in the log-log scale, which was used in the visual inspection. Straight lines with the slopes −1 or 0 were used in the age ranges [0, 365 days) and [1, 10) years, for preliminary assessment.

**Figure 3:**
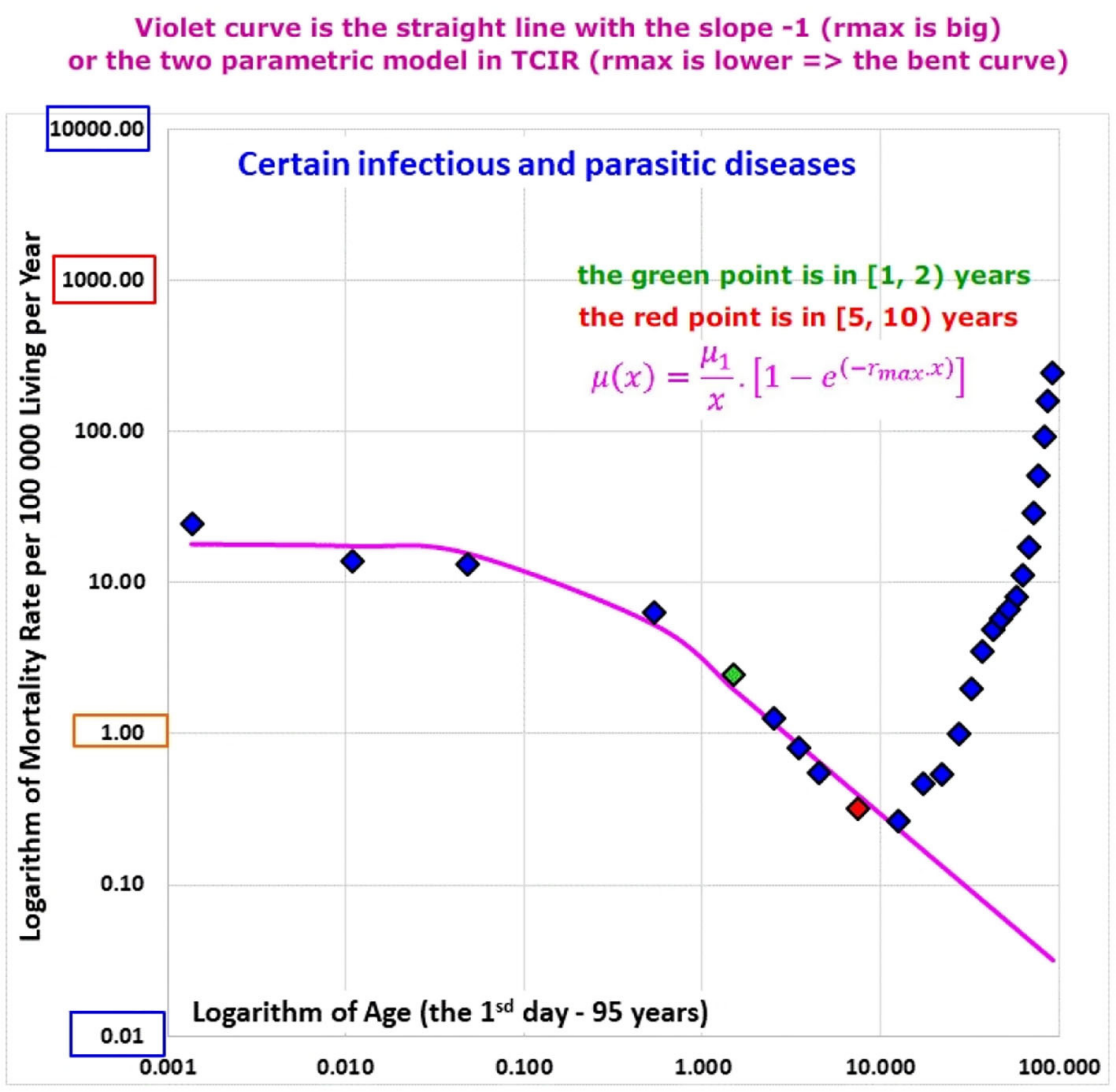
(Animation 2) Certain infectious and parasitic diseases in P14.

**Figure 4:**
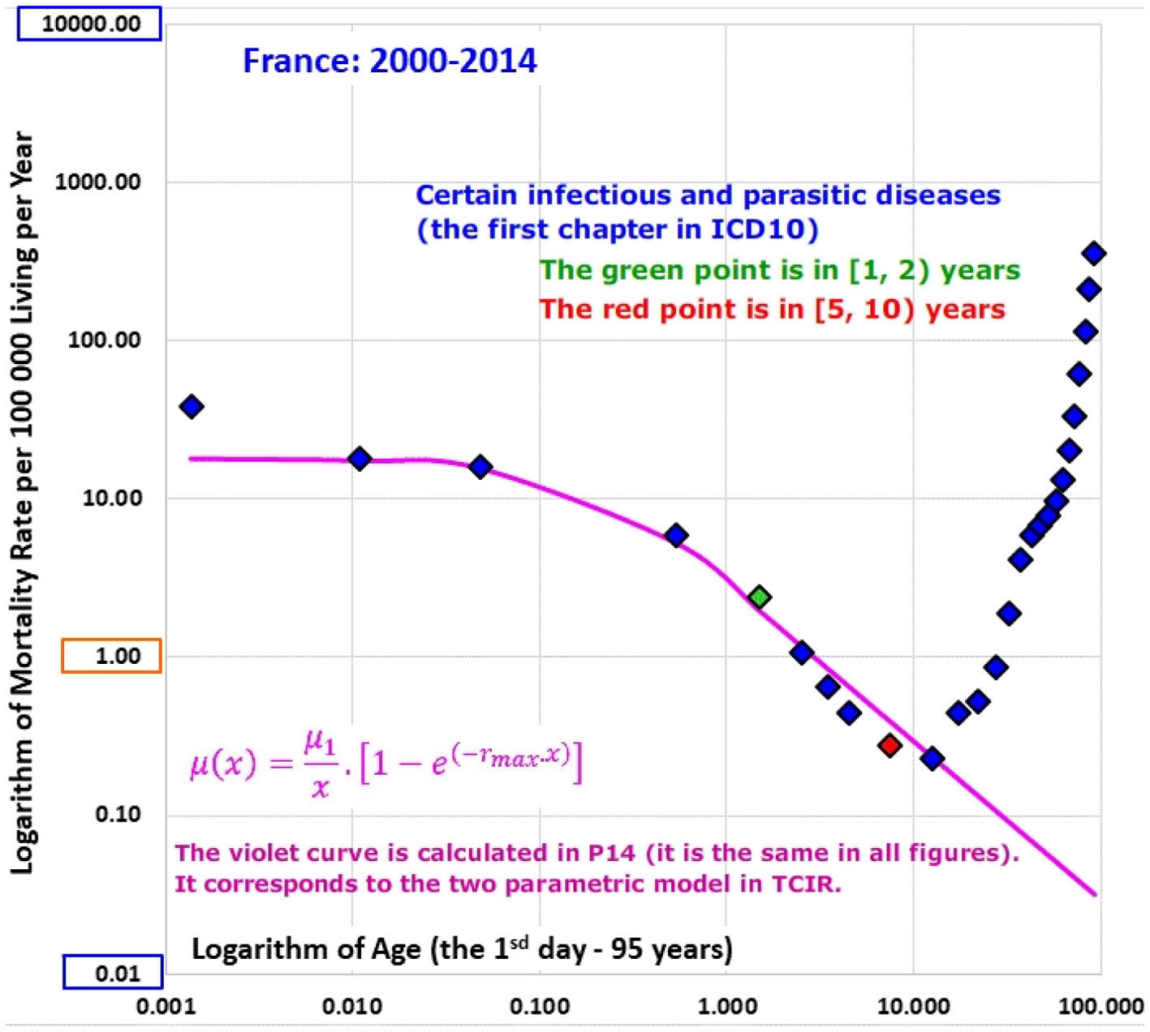
(Animation 3) Certain infectious and parasitic diseases in France.

ATM from diseases of the main ICD10 chapters [i.e., the chapters marked with “yes” for the P14 in the column “ATM” in Table 3, were constructed for each population (the first column of Table 2 shows the list of populations)]. ATM from accidents represents non-biological causes of death and were not investigated (this category comprised about 8% of deaths in P14 in the age range [0, 10) years). Initially, ATM were visually inspected in the log-log scale for all populations and the ATM of P14 are shown in Animation 2. The first ATM of Animation 2 is in Figure 3 [ATM from “Certain infectious and parasitic diseases” (Chapter I)]. ATM from “Certain infectious and parasitic diseases” (Chapter I) of all populations are shown in Animation 3 and the ATM of France is shown in Figure 4 (some of these ATM had undefined mortality rates within some age categories, due to the absence of death).

**Figure 5:**
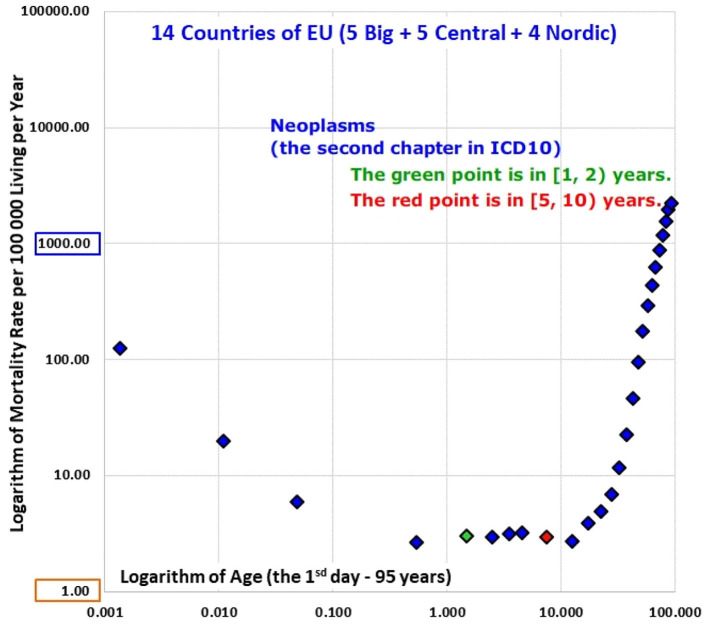
(Animation 4) Neoplasms in P14.

ATM from neoplasms (Chapter II) differed significantly form ATM of other ICD10 chapters. Results of all populations are shown in Animation 4 and ATM of P14 is shown in Figure 5. These ATM decreased during the 1st year of life but became age-independent from the 1st year to the 20th year of life. The decreased mortality rate from neoplasms during the 1st year may be described by the inverse proportion model (4) for all populations, except for Spain, U.K., Czech Republic, and Slovakia. The inverse proportion model (4) was not rejected in all other populations within the age interval [0, 365) days. All *p*-values calculated for neoplasms are shown in Table A1 in the Appendix.

Unlike neoplasms, mortality rates from other conditions had a faster decline after the 1st year of life than during the 1st year (see Animation 2). The mortality decrease was described by the inverse proportion (4) in six chapters within the age interval [1, 10) years in population P14 (the results are in the last column in Table 3), while the inverse proportion (4) was rejected only in two chapters of ICD10 in P14. A faster decrease in mortality from “Certain infectious and parasitic diseases” and a slower decrease in mortality from “Diseases of the genitourinary system” within the age range [1, 10) years were observed. More detailed figures are shown in Table 3. Age-independence (null hypothesis Ho: slope = 0) and the specific value of slope −1 were tested separately for the age ranges [0, 365) days and [1, 10) years. The bending ATM are also studied and discussed in the section “Theory of Congenital Individual Risks.”

**Figure 6:**
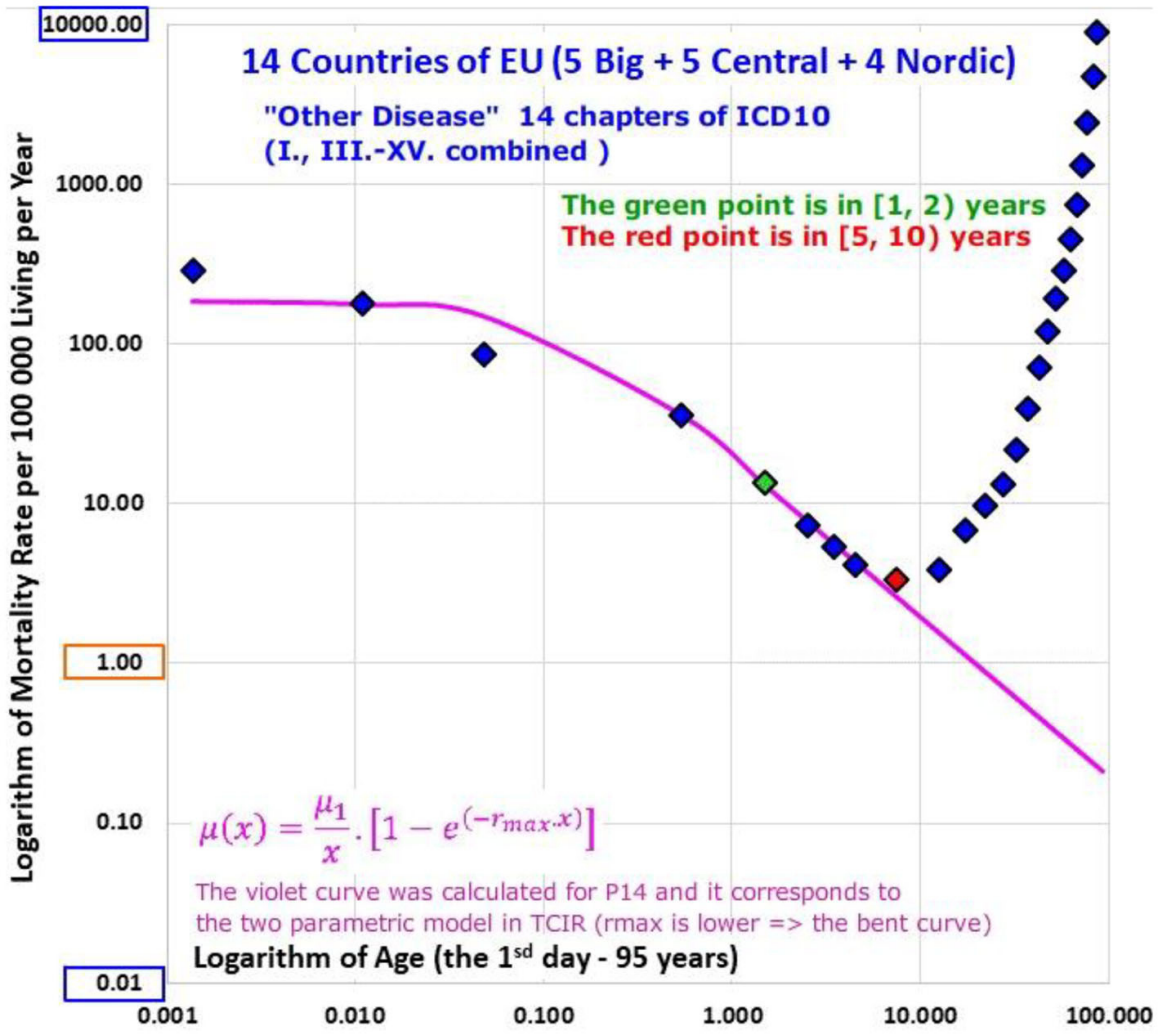
(Animation 5) Other diseases in P14.

ATM from conditions of chapters I, III, IV, VI, IX-XI, and XIV were bending in the log-log scale, and these causes of death are not related to congenital impairment (Animation 2). Diseases of all chapters for which mortality decrease was slower in the 1st year of life than after the 1st year, and were not related to congenital impairment were aggregated in the single group “Other diseases,” which comprised conditions of chapters I-XV without the second chapter “Neoplasms.” The results of ATM from “Other diseases” of all populations are shown in Animation 5 (ATM of P14 is shown in Figure 6).

### Age Trajectory of Mortality From Certain Conditions Originating in the Perinatal Period (Chapter XVI) and From Congenital Anomalies (Chapter XVII)

**Figure 7:**
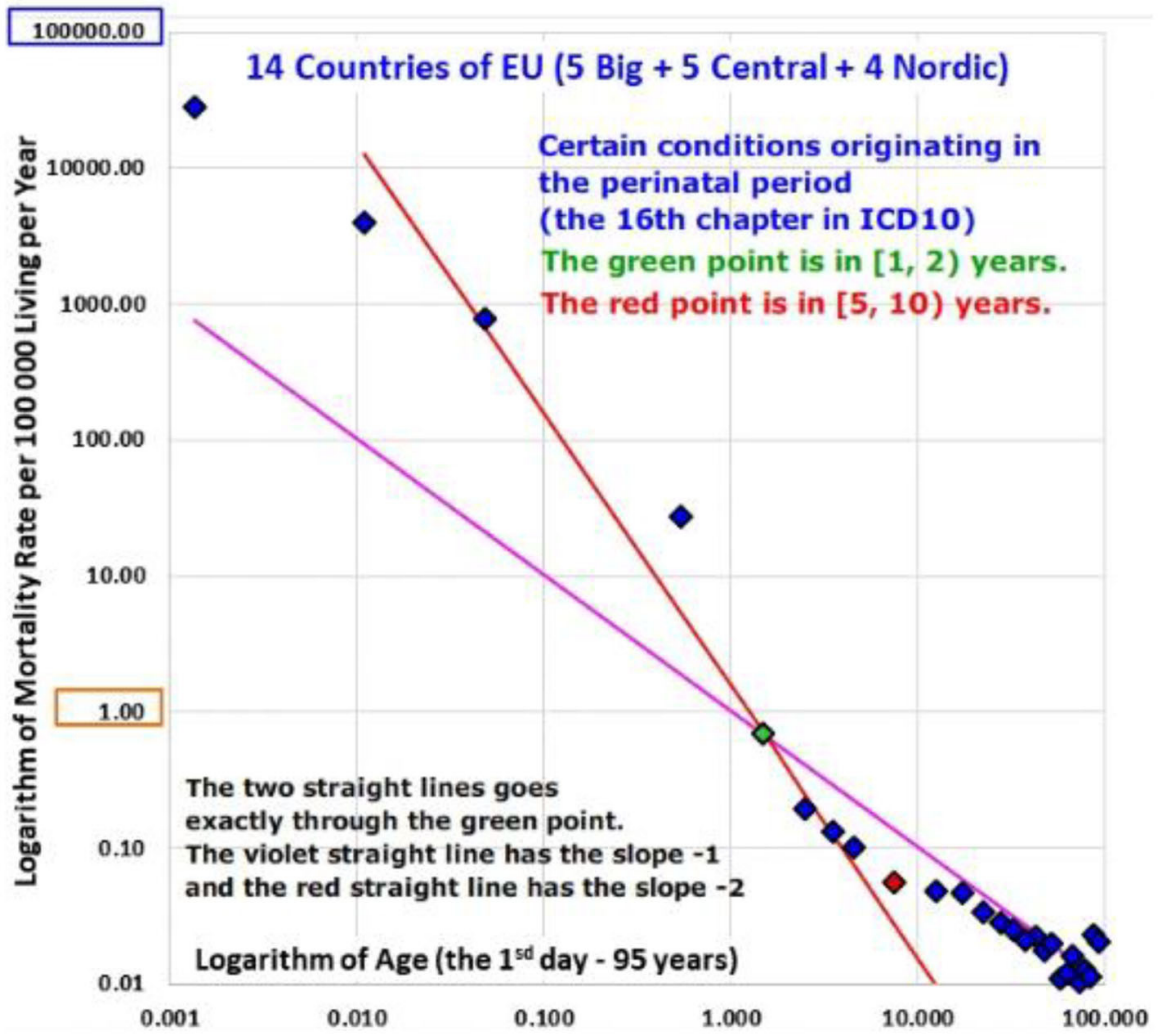
(Animation 6) Certain conditions originating in the perinatal period in P14.

**Figure 8:**
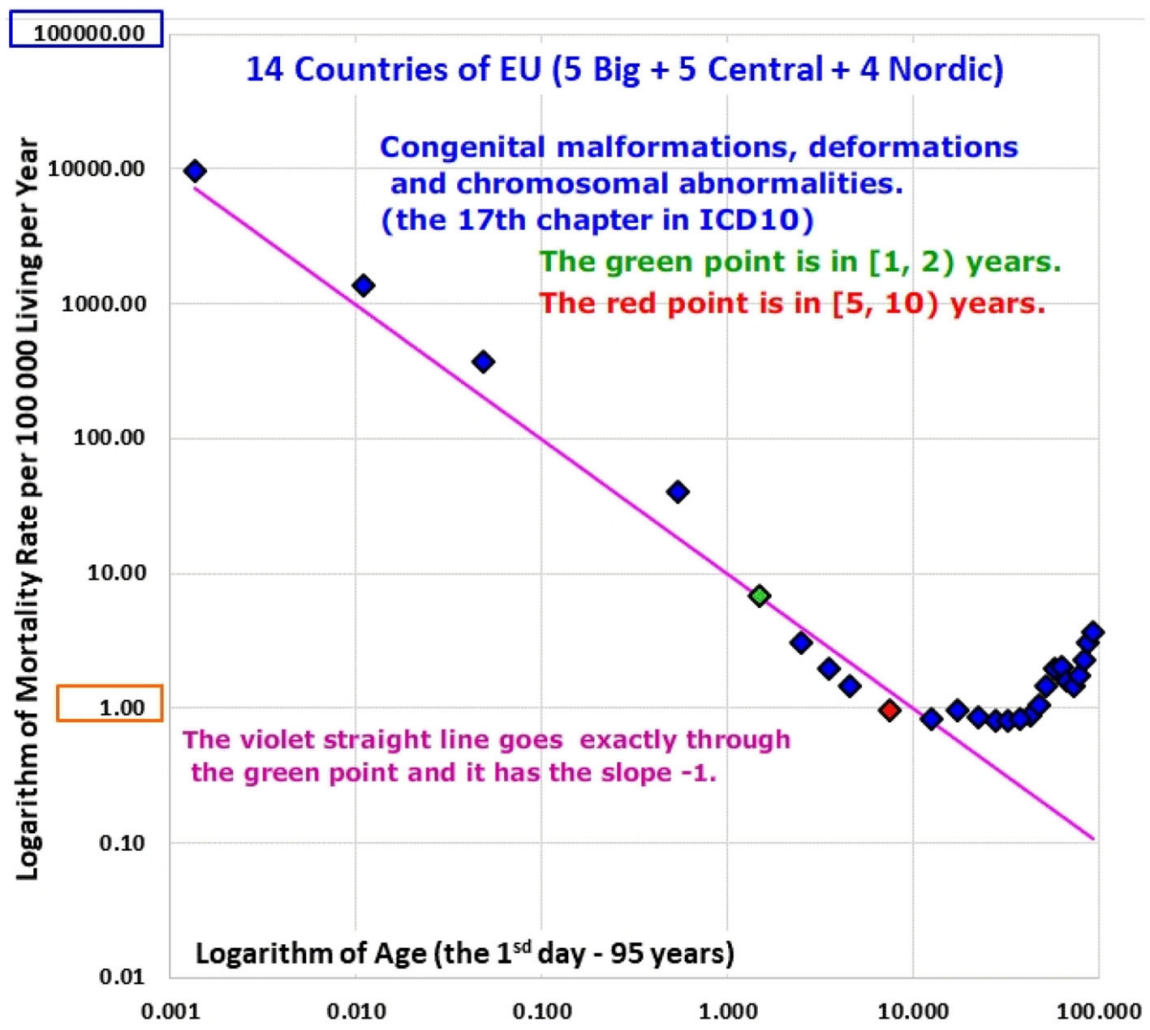
(Animation 7) Congenital malformations, deformations and chromosomal abnormalities in P14.

These two chapters differ from the other ICD10 chapters in a very important aspect: cases in the two chapters are related to congenital impairment or any impairment that was developed during the perinatal period. At first, ATM from these conditions were also visually inspected in the log-log scale; results of all populations are shown in Animations 6 and 7, and the ATM of P14 are shown in Figures 7, 8. No case was found in some age categories in some populations in the chapter “Certain conditions originating in the perinatal period” (XVI.). Consequently, 14 results of ATM from conditions of chapter XVI were available, while all 18 results of ATM from conditions of chapter XVII were available.

Bending ATM were not observed for conditions of the two chapters and ATM decreased to higher ages (see Animation 6 and 7). The shortest age range in which mortality decreased with age was [0, 15) years, and the age interval was used to the description of the mortality decrease unlike the inspection in the two age intervals in Table 3 (ATM decreased in the age range [0, 15) years also in P14).

Visual inspection of Animations 6 and 7 show that the variation in the linear model is larger for conditions of the chapter XVI and smaller for conditions of the chapter XVII. The linearity was rejected only in the U.K. and Poland, while it was not rejected in the rest 12 ATM in the chapter XVI (it means that the null hypothesis Ho: δ = 0 in the model (2) was tested and 14 ATM were obtained in the chapter). Regarding ATM from chapter XVII conditions, linearity was observed in all populations, except for those from Czech Republic, Slovakia, and Denmark.

In the next step, two specific slopes−1 and−2 in the log-log scale were tested within the age range [0, 15) years (only the linear ATM in the log-log scale were tested in the next step). The inverse proportion model (4) was rejected in all populations, regarding ATM from chapter XVI conditions (mortality decrease was steeper in the log-log scale). The specific value of−2 in the model (3) was accepted in three populations (France, Poland, and P2) but rejected in P14 and in the remaining populations. Interpretation and the meaning of the value−2 are discussed in the following text.

Different results were obtained for ATM from chapter XVII conditions (Congenital malformations, deformations and chromosomal abnormalities). The inverse proportion model (4) was not rejected in 12 out of 15 populations (the linearity was rejected in three populations). The inverse proportion model (4) was not rejected in the most important population P14.

In summary, the decrease in mortality from chapter XVI conditions with age was steeper, while the inverse proportion model (4) better fitted ATM from chapter XVII conditions (Animations 6 and 7). All detailed figures are shown in Tables A2, A3 in the Appendix.

### Age Trajectory of Mortality Due to Congenital Anomalies of the Central Nervous System (CACNS)

**Figure 9:**
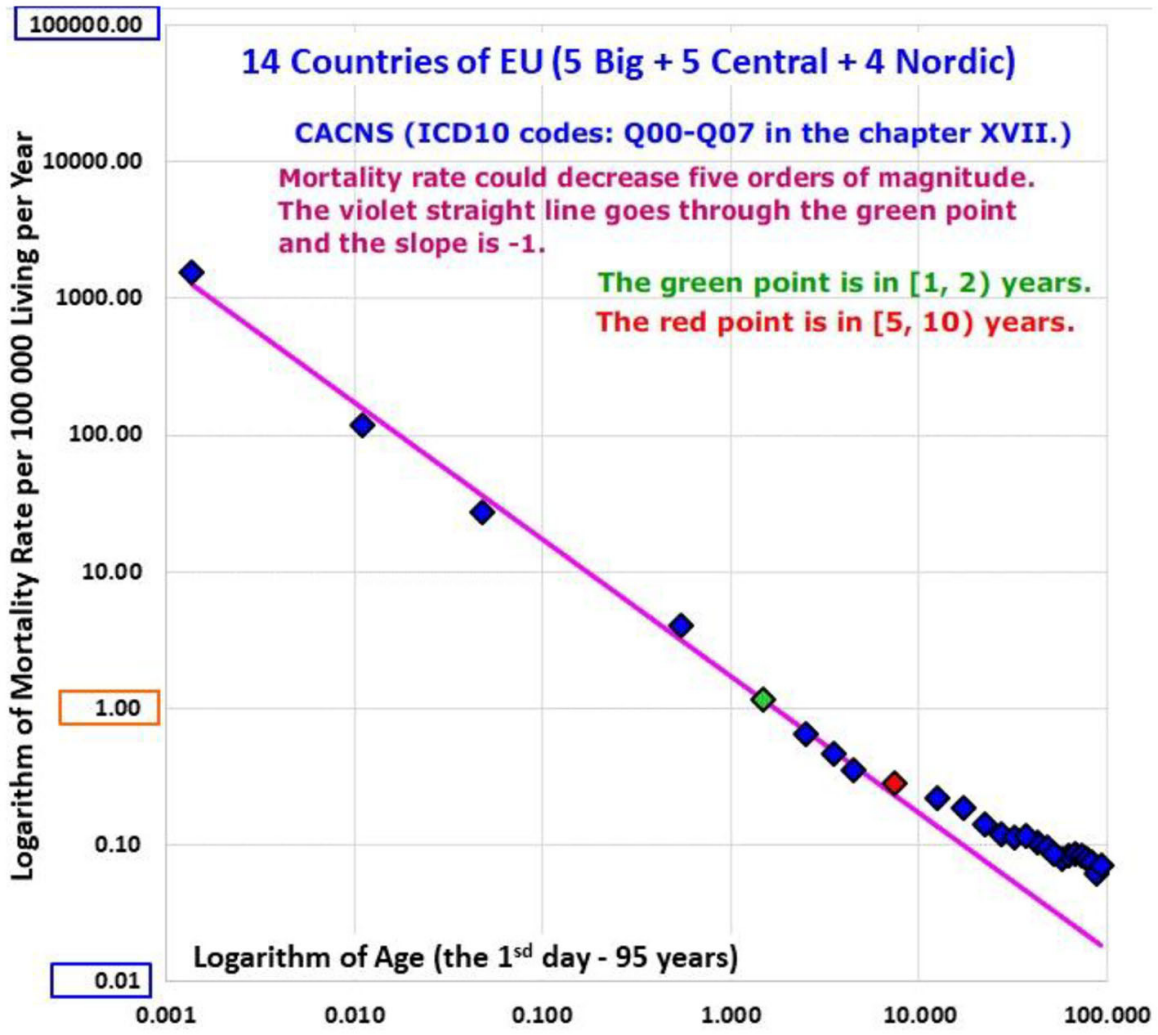
(Animation 8) Congenital anomalies of the central nervous system.

It was previously shown that the inverse proportion model was valid up to higher ages for congenital anomalies of the central nervous system (CACNS) with high coefficients of determination in other populations (26, 28). CACNS are designated by the Q00-Q07 codes in the ICD10 and were studied here as an individual disease group. Data on ATM from CACNS of all populations are shown in Animation 8, and the ATM of P14 is shown in Figure 9. The age range [0, 15) years was used to compare ATM from CACNS and from conditions of chapters XVI and XVII, as, in all populations studied, this was the lowest age range in which mortality decreased (results of the shorter age range [0, 10) years were very similar). All 18 ATM due to CACNS were available in the age range [0, 15) years.

Linearity was rejected in six countries (Germany, Italy, Austria, Poland, Slovakia and Finland), while it was not rejected in all other countries and in all aggregated populations P1, P2, P3, and P14. Consequently, the inverse proportion model (4) was tested in 12 populations and it was rejected only in Czech Republic and Hungary. The coefficients of determination calculated in the model (4) were very high, in general, reaching the maximum value of 0.9964 in P1, and the value of 0.9958 in P14. All these results are shown in Table A4 in the Appendix. Arithmetic mean of ${\text{R}}_{\text{b}}^{2}$ calculated for 9 ATM in the age range [0, 15) years was 0.9868 (standard deviation = 0.0078).

The coefficients of determination ${\text{R}}_{\text{b}}^{2}$ calculated for ATM from CACNS in the model (4) were almost as high as those calculated for total mortality (Table 2). For example, the coefficient of determination was 0.9967, for total mortality within the age range [0, 10) years in P14, while a value of 0.9958 for ATM from CACNS was found in P14 in the same age range. The inverse proportion model (4) was not rejected in 12 populations in the age range [0, 10) years. Arithmetic mean of ${\text{R}}_{\text{b}}^{2}$ calculated for 12 ATM from CACNS in the age range [0, 10) years was 0.9873 with standard deviation 0.0076.

Overall, the mortality decrease with age was slower within age ranges above 20 years in some populations (see Animation 8).

### Age Trajectory of Mortality Due to Symptoms, Signs, and Abnormal Clinical and Laboratory Findings, Not Elsewhere Classified (Chapter XVIII)

A bending ATM was also detected for conditions of chapter “Symptoms, signs and abnormal clinical and laboratory findings, not elsewhere classified” (XVIII), which contains cases with problematic diagnosis. ATM in P14 was age-independent in the 1st year of life and decreased within the age range [1, 10), according to the inverse proportion model (4) (Table 3). A total of 7.88% of all deaths due to these conditions in P14 occurred within the age range [0, 10) years.

### Theory of Congenital Individual Risks (TCIR)

The previous results may be explained by the theory that mortality decrease with age is caused by the extinction of individuals with severe congenital impairment. This theory is based on the assumption that some children had a congenital impairment, and this was approximately age-independent. The majority of the population had a very mild impairment or none impairment, which was not relevant to the ATM calculation. Age-independence is a rough approximation, and it is used to describe heterogeneity in the whole population. The main reason for the aforementioned assumption is that the decrease in the mortality rate from CACNS with age is possibly caused by the extinction of severe impairments. In addition, the decrease in mortality from CACNS with age was previously indicated by the inverse proportion model (25, 26, 28). Herein, total mortality also decreases with age, according to the same model, with high coefficients of determination (Table 2). In fact, a congenital impairments leading to a congenital individual risk of death may change with age. Such changes are not addressed here, considering the whole spectrum of impairments in the born population. Simultaneously, the majority of the population has very small or absent congenital individual risks of death, which render them irrelevant to ATM.

A population is composed of subpopulations characterized by specific impairment levels, and each congenital impairment level is described by a continuous value (*r*), which corresponds to the congenital risk of death or mortality rate per one person-year. It is an empirically unknown value for concrete person and, furthermore, some distribution of r may be assumed in born population. In other words, it is assumed that the shape of ATM is a hallmark of the *r* value distribution at the moment of birth. Survival function S(r, x) in the subpopulation of individuals with similar impairments and with the same value of r is:

(7) $$\begin{array}{l} S\left(r,x\right)=\text{exp}\left(-r.x\right) \end{array}$$

This is probably the simplest survival curve and it is also valid for the proportion of specific radionuclide in time x (if the r value in the formula (7) is replaced by the decay constant). Derivation of relationships between mortality rate and x is presented in the appendix in the first paragraph Equations in TCIR.

If it is assumed that “the more severe the impairment, the less frequently it occurs in the born population,” mortality rate decreases with age according to the inverse proportion model (4) and the Equation (15) in the appendix is valid. Such distribution of r may be explained by the biological selection of higher values of r, which could proceed during the previous generations or during pregnancy.

The interpretation of the formula (16) in the appendix is: “if the incidence of different *r* values is approximately the same,” the slope of mortality decrease with age in the log-log scale is about−2. For example, such situation may happen after a huge catastrophe. In a more general way, it may happen when the selection of serious impairments is not present. This may be approximately valid for impairments developed in the perinatal period, which are grouped within the chapter “Certain conditions originating in the perinatal period (XVI).” This is a possible explanation for the steeper decline in mortality due to chapter XVI conditions with age.

### Bending Age Trajectory of Mortality

Bending ATM represent the next empirical verification of TCIR (see Animations 2, 3, and 5). At first, it is difficult to assume that any hypothetical individual development is more significant after the age of 1 or 2 years (it has to be valid if any homogenous description of population is assumed). Such hypothetical homogenous development has to be significantly slowed down during the 1st year and it is more significant after the 2nd year up to the age of 10 years.

In contrast, the TCIR explains the mortality decrease with age based on the sequential extinction of more severe impairments, and it could also explain the bending ATM. Mathematically, if maximal value r_max_ of congenital individual risks in the born population is not big and infinity is replace by r_max_ in Equation (13) in the appendix, the following approximation is valid (25, 26):

(8) $$\begin{array}{l} \mu \left(x\right)=\underset{0}{\overset{r_{max}}{\int }}c.e^{\left(-r.x\right)}dr=c.{\left[\frac{e^{\left(-r.x\right)}}{-x}\right]}_{0}^{r_{max}}=c.\frac{e^{\left(-r_{max}.x\right)}}{-x}-c.\frac{1}{-x}\\ \;=\frac{\mu_{1}}{x}.\left[1-e^{\left(-r_{max}.x\right)}\right] \end{array}$$

Furthermore, if the product *r*_max_. *x* is smaller than 1 in Equation (8), the theoretical mortality rate is approximately constant. It follows from the approximation (9) (25, 26):

(9) $$\begin{array}{l} \mu \left(x\right)=\frac{\mu_{1}}{x}.\left[1-e^{\left(-r_{max}.x\right)}\right]≅\frac{\mu_{1}}{x}\left[1-\left(1-r_{max}.x\right)\right]=\mu_{1}.r_{max} \end{array}$$

If a time unit of 1 year is used, and if r is per year, then the product r_max_.x is unit less. The approximation (9) is valid if the product r_max_.x is smaller than 1. On the other hand, if age x is higher (x > 2), the exponential term in the squared brackets can be neglected in Equation (8), and the inverse proportion (15) in the appendix is valid. Consequently, the theoretical relationship (8) may explain the bending ATM, and it was used to fit bending ATM.

Why may congenital risks be limited in some categories of diseases and why may the limit be higher for congenital anomalies? For example, if an individual case of death is related to infectious disease and no visible congenital impairment is determined, a hypothetical congenital impairment is latent. Some ICD10 chapters do not automatically contain cases with severe and visible congenital impairments (unless such impairments exist). If TCIR is valid for diseases that are not related to congenital anomalies, the spectrum of r should be limited by relatively small value of r_max_ for the diseases. This explanation may account for the empirical ATM in Animations 2, 3, and 5, or in Figures 3, 4, 6.

For these reasons, the model (8) was used here and the two parameters r_max_ and μ_1_ were estimated. The two values were numerically calculated by minimizing the residual sum of squares with the coefficients of determination. The values for P14 are shown in the two last columns in Table A5 in the Appendix.

On the other hand, no such small limit of r may exist in the all-cause category, and this is the explanation for why ATM were not bending and the model (4) fits them in the whole age interval [0, 10) years (Table 2). The limit r_max_ may correspond to a medical definition of a specific ICD10 chapter and may also differ according to the country. The relationship (8) could explain the decrease in the total mortality rate with age, according to an inverse proportion following the 1st day of life, which may be age-independent during the 1st year of life.

As higher the r_max_ the straighter the ATM is in formula (8) in the log-log scale (empirically, the highest value of r_max_ was in the chapter XVIII. in Table A1). It should be emphasized that the inverse proportion (4) was valid for total mortality from the 1st day of life and the coefficients of determination in Table 2 were very high.

As the bending ATM may be explained by TCIR for conditions of all chapters with the bending ATM were grouped in one category of diseases and the category is labeled as “Other diseases.” The category “Other diseases” does not contain Neoplasms (II) and the two chapters XVI. and XVII. related to some impairment present after the perinatal period (this set of chapters contained the chapters I and III–XV). The results calculated for the category “Other diseases” are shown in the last row of Table A5 in the Appendix.

The most important coefficients of determination ${\text{R}}_{\text{b}}^{2}$ calculated for P14 with in the age interval [0, 10) years in the model (4) are: 0.9967 (total mortality), 0.9958 (CACNS), 0.9815 (the chapter XVII), and adjusted coefficients of determination calculated by the model (15) for the bending ATM are shown in the last column of Table A5 in the Appendix. The highest values of the bending model (15) were 0.977 for conditions of the first ICD10 chapter and 0.968 for conditions of the “Other diseases” category. The results are also shown visually in Animations 1, 5, and 8.

All the results show that the possible mechanisms responsible for the model of the inverse proportion (4) may be general biological process. The values of ${\text{R}}_{\text{b}}^{2}$ were very high for CACNS, where the mechanisms were well-visible and where the extinction of more severe impairments is obvious.

### Other Historical Models of Age Trajectories of Total Mortality After Birth

**Figure 10:**
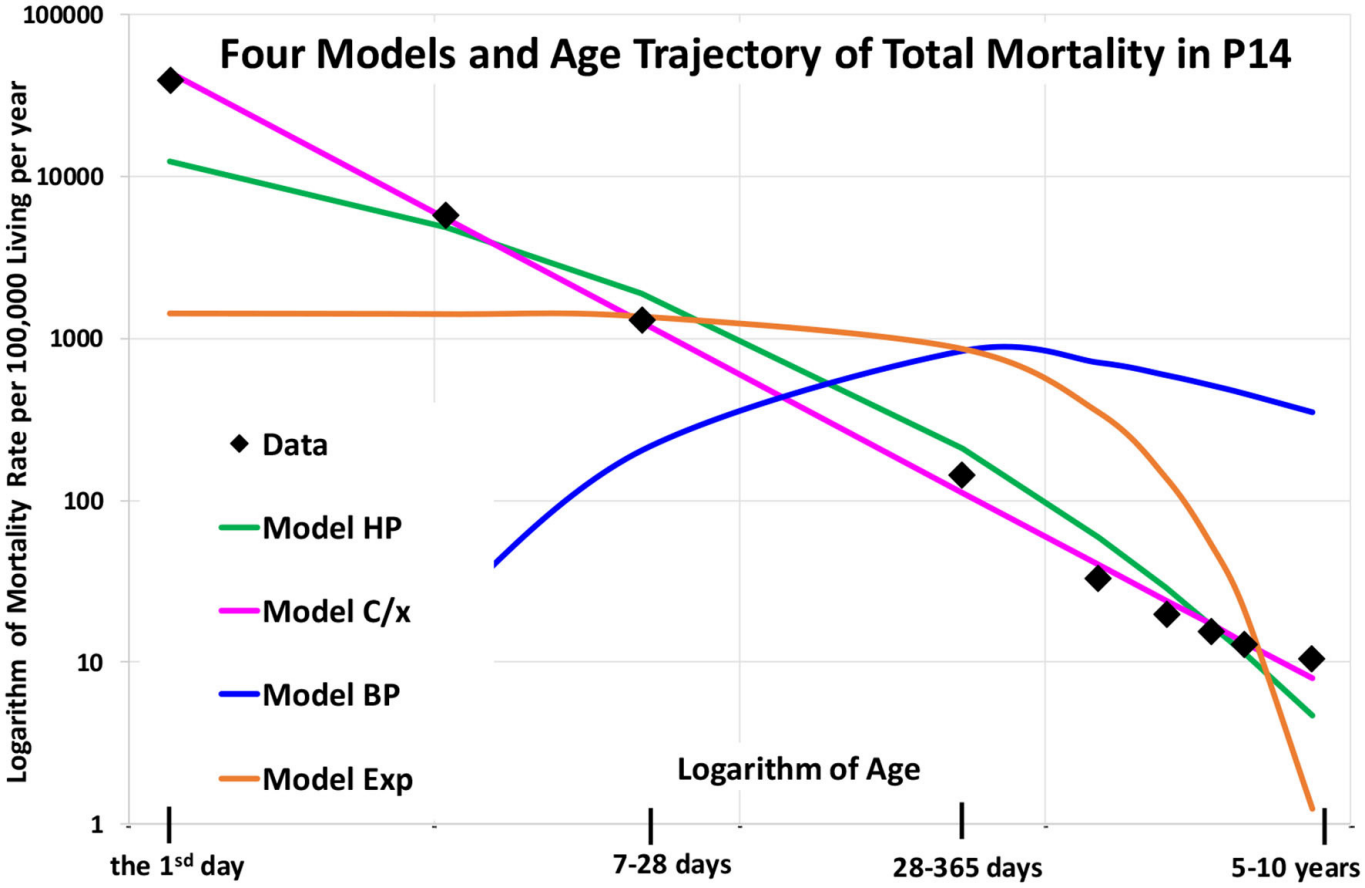
(Animation 9) Four historical models and age trajectory of total mortality in P14.

The list of all parametrical attempts to describe age trajectories of human mortality after birth is not long. Four models, which are described in the appendix, were published in the scientific literature. Their ability to fit nine age categories in the age interval [0, 10) years, which were used in the WHO database, is not high. Age trajectory of total mortality in P14 was fitted in the present study using these models and the LS method. The resulting curves are shown in Figure 10 and the inverse proportion model (4) fits data preferably. The adjusted coefficient of determination was 0.9967 in the inverse proportion model (4), 0.9396 in the HP model, 0.4241 in the Exp model, and was negative in the BP model (the sum of squares was bigger than in the trivial model without parameters).

Consequently, the ability of models Exp and BP to explain the data is very weak (model BP is even increasing in lower ages). Model HP showed a greater ability, but the coefficient of determination is much lower (0.9396), when compared with the inverse proportion method (0.9967). Besides, model HP is concave in the log-log scale and linearity was not rejected in all populations, regarding total mortality. The parameter δ in the model (2) may closely estimate the ATM curvature in the log-log scale. Its estimation was positive in seven countries, and negative in other populations, including P1, P2, P3, and P14 (statistically, the null hypothesis Ho. δ = 0 was not rejected in all populations). The results show that the models Exp, BP, and HP were not suitable to describe age trajectories of total mortality after birth.

The Weibull model (WM) with two parameters is linear in the log-log scale and may be identified with the model (3). It may fit linear data in the log-log scale but absolute value of the slope of mortality decrease in the log-log scale should be <1 (the slope has to be > −1). Consequently, WM may not fit steeper decrease with slope equals to−1 or < −1. WM is identical with the linear model (3) if slope is > −1. Because the slope calculated in WM was very close to-1 (the estimation calculated in WM was −0,9964) the straight line calculated in the model (3) and the straight line calculated in the model (4) are graphically identical and both models are represented by one line in Figure 10.

## Discussion

The relationship between mortality rate and age was the main finding in the present study and shapes of ATM were used for these evaluations. Generally, shapes come from the world of platonic ideas according to the Greek “theory of forms,” and for example, no physical object has, at first, the shape of a perfect circle. Statistically, proofing a linear relationship is not possible, but it may be rejected. Likewise, it is not possible to distinguish the two values of slope−1 and −1.000001 in the linear model. The value−1 is simply assumed because it may be interpreted easily. On the other hand, if the percentage of an explained variation is bigger than 0.99 or even bigger than 0.998 then such model is very successful. It was observed here and the empirical relationships were very deterministic. Such values of coefficients of determination were observed for total mortality and CACNS mortality.

The log-log scale was used for two reasons. At first, the shape was more visible in this scale and, secondly, residuals were not dependent on the explanatory variable, which was the logarithm of age (the basic assumptions of regression model was not satisfied in the standard scale). The inverse proportion model (4) has one parameter and the estimation of the parameter has the same degrees of freedom (n−1) as the standard estimation of the arithmetic mean, and the only parameter may be estimated using the formula (5).

### General Look at Change Over a Period of Time

There are two different ways to the description of change in time. The first possibility is to express the change regardless of the original value of an observed parameter. The second possibility is to relate the change to the original value. The two ways represent the background of the difference between intensive and extensive quantities. For example, if the weight of an individual is increased by 5 kg, the value of 5 kg represents the first possibility. If the baseline individual's weight was 50 kg, for instance, the second possibility is to state that the weight had a 10% increase. If the baseline weight was 100 kg, the increase was 5%. The first way was primarily chosen in mathematics in the definition of derivation (value of derivation does not depend on the original value of the function but only on the function's “shape” or “behavior”). The value of the derivation of the function divided by the original value of the function represents the second way, and it is one interpretation of the definition (1). Regardless of the use of S(x) or L(x) in the equation (1), the changes are expressed in relation to the original values. If the formula (1) (the mathematical operator) is applied on ATM once more, interesting results are obtained. If it is applied on the standard Gompertz formula, a simple constant is obtained. The well-known exponential increase of mortality with age is valid for adults (4, 18, 19, 21, 22) The resulting constant is the slope of the linear mortality increase in the semi-logarithmic scale (one of the two Gompertz parameters). It is probably the more important parameter describing the shape of ATM (the dynamics of the increase in mortality of adults with age). If the formula (1) is applied to the inverse proportion model (4), a simple inverse proportion “1/x” without any parameter is obtained. The two curiosities may have been important to the interpretation of ATM in all ages and to general mechanisms responsible for ATM constructed for all ages.

### Two Kinds of Time

If the change in time is related to living individuals, two kinds of time are usually used. They may be labeled as “inner time” and “outer time.” Standard Lexis diagram applied to demography is one example of the use of the two kinds of time. Calendar year is considered outer time in Lexis diagram, while age is considered inner time. Usually, the two kinds of time are not distinguished in science. If the outer time is used, the onset of time axis usually has a different meaning, if compared to the use of the inner time. Zero has different meaning in different calendar systems, and, usually, the onset is not important. It may be situated to the first Greek Olympic games or to any other time point. If ATM are studied, the birth or the onset of time axis has a more important role, and the onset should not be arbitrarily defined. For example, based on these considerations, age-independence of ATM during the 1st year and within the age range [1, 20) years have different interpretations. If the onset of time axis has a crucial meaning (e.g., the birth), the “inner time” is used and change in time means that the change proceeds inside the system. For example, ATM of a given population corresponds to “inner time” and correspond to the change proceeds inside the system. On the other hand, physical trajectories of the same individuals correspond to “outer time” (trajectories describing the movement of studied people during their life) and correspond to the change proceeds outside the system. Such considerations may explain the use of the logarithmic scale for describing ATM because the changes corresponds to inner time.

### Halley Method

The relationship between mortality and age had a crucial meaning here and it was the first reason to construct ATM using the Halley method in P14 (impact of age may be more visible in shapes of the ATM) (38–40). The second reason to use the method was the fact that no deaths from some diseases were detected in some calendar years.

The assumption that some general mechanisms exist, which are responsible for the mortality decrease with age after birth, was strongly confirmed (see Animations 1–8). Regardless of TCIR, inverse proportion model (4) is dominant through the whole spectrum of diseases and it should be a hallmark of some biological mechanisms. Observations also supported the statement that ATM constructed for the large populations P1, P2, P3, and P14 were smoother, and the coefficients of determination were higher in these populations, for total mortality and for mortality from CACNS (Table 2, Table A4).

Generally, the determination of cause of death may differ within 14 studied countries and it was not clear if the same case was put in the same subcategory of ICD10. It was assumed here that such uncertainty was less probable for the main chapters of ICD10 because they were relatively extensive. Besides, it was empirically confirmed here that the shapes of ATM were almost identical in studied 14 European countries.

### Extinction of Individuals With More Severe Impairments

The explanation of the steep decrease of mortality with age represented the second viewpoint had mentioned in the introduction. The assumption of TCIR that the decrease in mortality with age was caused by the extinction of individuals with more severe impairments may be indirectly supported by the two following results:

1. Total mortality rates decrease according to the inverse proportion model (4) and, simultaneously, the significance of the two chapters “Certain conditions originating in the perinatal period” (XVI.) and “Congenital malformations, deformations and chromosomal abnormalities” (XVII.) decrease dramatically with age in Figure 1.
2. The CACNS category contains the following eight subcategories: Anencephaly and similar malformations (Q00); Encephalocele (Q01); Microcephaly (Q02); Congenital hydrocephalus (Q03); Other congenital malformations of the brain (Q04); Spina bifida (Q05); Other congenital malformations of spinal cord (Q06); Other congenital malformations of nervous system (Q07). ATM from CACNS showed higher coefficients of determination and the ATM decreases up higher ages. The proportions of specific subcategories of CACNS are shown in Table A6 in the Appendix, and the proportions of Q00, which includes more severe congenital anomalies, strongly decrease with age.

Extinction of individuals with more severe impairments is acceptable during the 1st year of life and it is not self-evident in higher ages. Unfortunately, the incidence of congenital anomalies among living people were usually registered only at the moment of birth (27, 43). The incidence of congenital anomalies after birth is difficult to find in scientific literature, and only the proportions of congenital anomalies among individuals who died is available. The assumption that mortality decrease with age is caused by the extinction of individuals with more severe impairments is supported by the results found for ATM from congenital anomalies.

The main problem of explaining the ATM shapes using any homogenous process, which is assumed to be valid for each person, is acceptance of such explanation for CACNS and other congenital anomalies. The shapes of ATM due to CACNS and congenital anomalies were the same in the 1st years and after the 1st year of life. Besides, ATM due to CACNS was described by the inverse proportion model with a single parameter from birth up higher ages. In addition, such hypothetical mechanisms should be suppressed during the 1st year and it should cause faster mortality decrease in the age interval [1, 10) years for diseases grouped in the category “Other diseases.” Simultaneously, the mechanisms is in operation in the two chapters “Certain conditions originating in the perinatal period” (XVI) and “Congenital malformations, deformations, and chromosomal abnormalities” (XVII) form birth up to higher ages. If homogenous process is assumed then the fact that the inverse proportion is valid for the group “Other diseases” within the age range [1, 10) years and for congenital anomalies within the age range [0, 10) years is a random coincidence (see Animations 5, 7, and 8). Consequently, hypothesis that the shapes of ATM were due to any development of each individual is difficult to accept. An alternative explanation of the observed results is that the shapes of ATM were caused by the extinction of individuals with more severe impairments.

### Latent Congenital Risks of Death

If TCIR is correct, significant part of the deaths within the age interval [0, 10) years was, in fact, caused by congenital abnormalities (congenital risk of death), even in the cases registered as “Other diseases.” For example, if such individuals were affected by an infectious disease and died, the deaths may have been caused by congenital latent impairment. Most deaths until age 10 years may have been caused by these latent impairments, and the figures in Table 3 provide simple estimations about the significance of the latent congenital impairments. For example, 16.4% of the deaths until the age of 10 years were due to conditions grouped in the category “Other diseases,” and, simultaneously, ATM in this category was bent. According to TCIR, most cases may be caused by latent congenital impairment. If TCIR is correct, the shapes of ATM and the extinction of more sever congenital impairments may be consequence of the interaction between born population and current living conditions.

### Neoplasms

Other results were observed for conditions of the chapter “Neoplasms” (II). Mortality decrease with age occurred in the 1st year of life and was age-independent within the age range [1, 20) years. It may be important for the verification of any hypothesis that a specific type of neoplasm is only significant to a subpopulation with congenital predisposition (e.g., a genetically susceptible subpopulation). According to TCIR, such subpopulation should be larger than the sum of all deaths in the age interval [1, 20) years, since no extinction was observed. The sum of all deaths within the age range [1, 20) years represents the lower limit of the subpopulation assumed to be susceptible to the disease. This suggested limit is based on all ATM results and is very easy to be calculated. On the other hand, such number is only the lower limit of the hypothetical subpopulation. This means that if any predisposition exists then considered disease has to relate subpopulation large than the sum of all death in the whole specific population. For example, the proportion of all deaths due to conditions of the chapter “Neoplasms” within all living people in P14 was about 0.00003, which means that the incidence of these conditions in a hypothetical subpopulation should be higher than 3 per 100,000 living people. The result is not very significant, as it concerns the whole chapter “Neoplasms” and the proportion is relatively small. If very specific diseases of the chapter “Neoplasms” are analyzed, the age-independence may be empirically confirmed in the first step. It is not necessary to construct ATM for the age range [1, 20) years (no deaths may occur within a specific age range, even in P14). The age range [1, 20) years may be divided into two intervals only, and approximately age-independence may be tested in the two values. The number of all deaths represents thereafter the lower limit of the hypothetical subpopulation.

## Conclusions

The coincidence between All-causes ATM and ATM constructed for congenital anomalies (or ATM constructed for CACNS) was confirmed in all populations. The model of inverse proportion was found as a general rule. The bending ATM was also described by the inverse proportion behind the age of 1 year (it was age independent or decreased slowly during the 1st year of life). Consequently, child mortality decrease with age may be the result of the sequential exclusion of individuals with more severe impairments. The opposite explanation that homogenous development of every individual was responsible to the decrease was suppressed here.

All results are based on the WHO mortality database (41) and the analysis covers 369,865 deaths at the age range [0, 10) years in P14. The division of the 1st year of life into four age categories is crucial for these observations, and all results are based on published data (39). The sums of death numbers from diseases of all analyzed chapters, within all populations and within all age categories are presented here as a supplement. Sums of living individuals within specific age categories and within specific populations are also shown in a supplement. The sums may be recalculated using the WHO mortality database and all ATM may be verified in the second step (41). The Halley method enables the expansion of the data used in the present study to more countries in the future (38–40). Verification of TCIR for congenital anomalies may be performed by using any register containing incidence of congenital anomalies within more age categories. It may confirm or reject the assumption that the extinction of more severe cases is significant. If TCIR is valid, latent congenital impairments should be present among the deaths from conditions registered in the group “Other diseases” and/or from specific diseases of the group. The latent impairments may be detected among dead from some disease and simultaneously, are not among surviving the same diseases. Consequently, if the detection is successful, the knowledge about latent impairments may help to lower mortality from these diseases.

## Data Availability Statement

Publicly available datasets were analyzed in this study. This data can be found here: https://www.who.int/healthinfo/statistics/mortality_rawdata/en/.

## Author Contributions

JD realized statistical computations and composed the main text. HH construct and write all parts related to pediatrics and clinical interpretations. All authors contributed to the article and approved the submitted version.

## Conflict of Interest

The authors declare that the research was conducted in the absence of any commercial or financial relationships that could be construed as a potential conflict of interest.
