# Supplementary figures and images for: Why Does Child Mortality Decrease With Age? Modeling the Age-Associated Decrease in Mortality Rate Using WHO Metadata From 14 European Countries
Source: Front Pediatr. 2020 Oct 27;8:527811. doi: 10.3389/fped.2020.527811 (PMC7653179; doi:10.3389/fped.2020.527811)

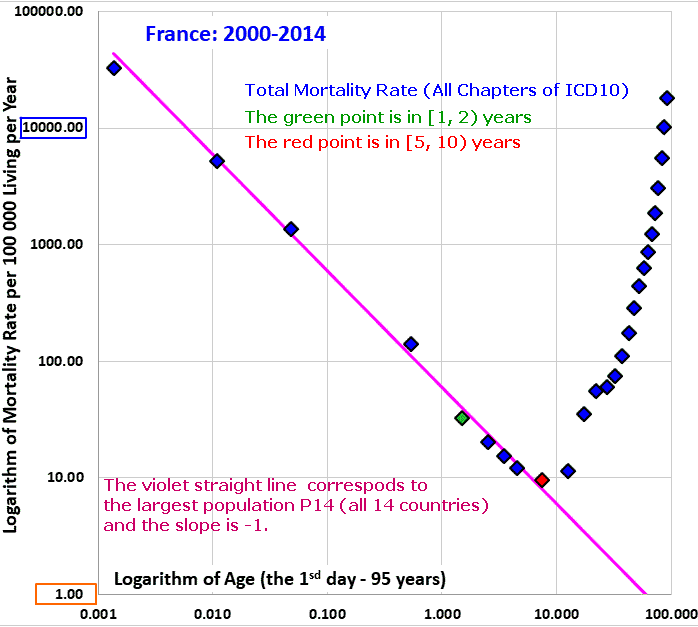

Supplement: Supplementary file 2 [file Data_Sheet_1.ZIP › Animation 1.gif]

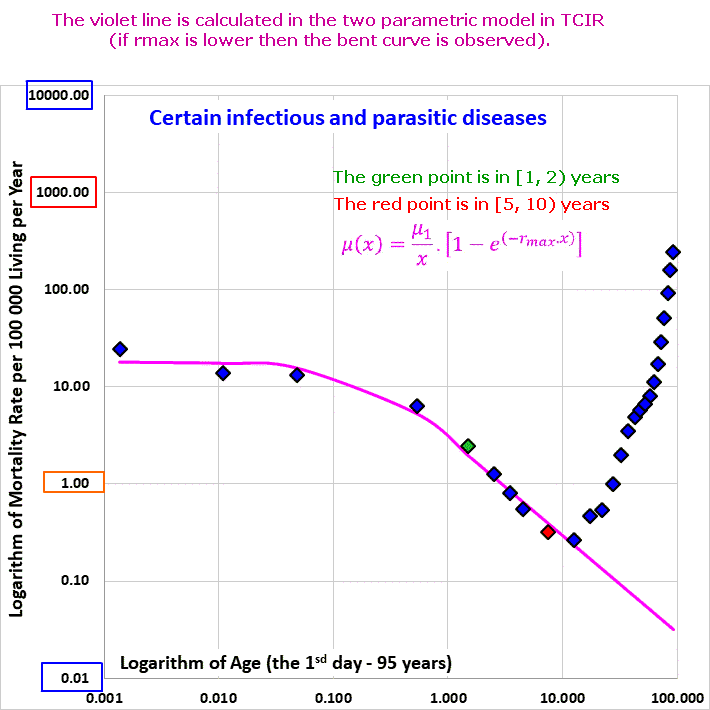

Supplement: Supplementary file 2 [file Data_Sheet_1.ZIP › Animation 2.gif]

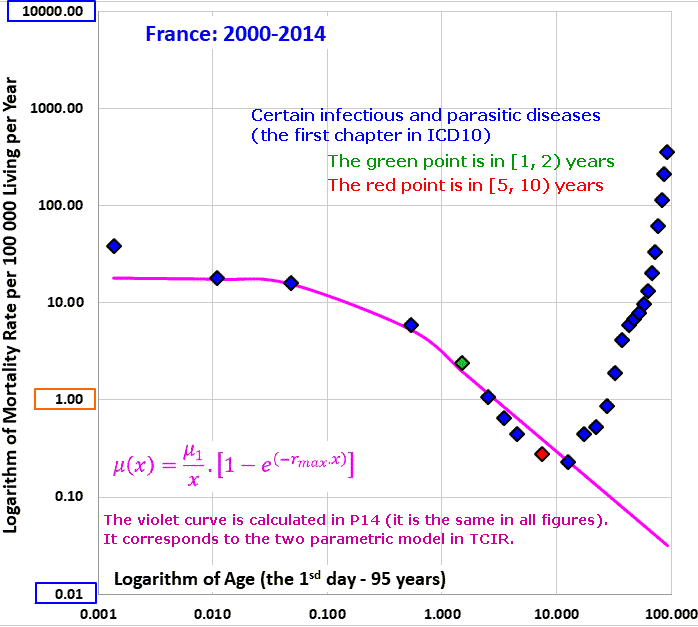

Supplement: Supplementary file 2 [file Data_Sheet_1.ZIP › Animation 3.gif]

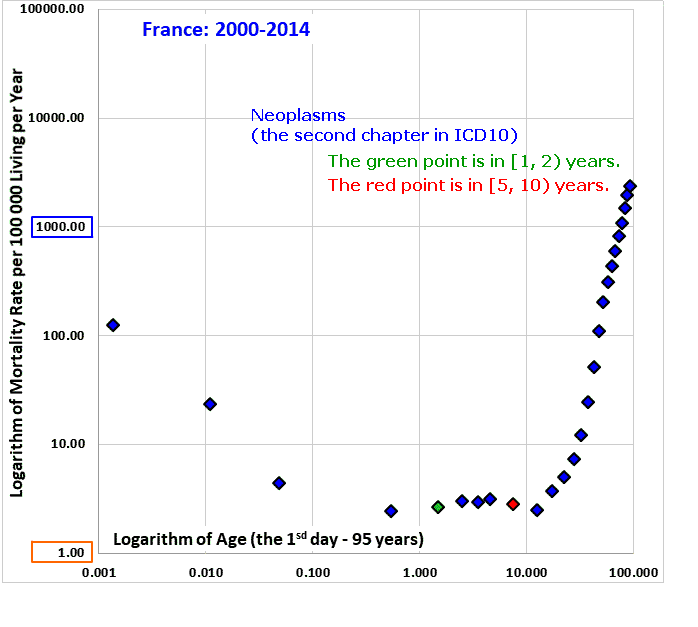

Supplement: Supplementary file 2 [file Data_Sheet_1.ZIP › Animation 4.gif]

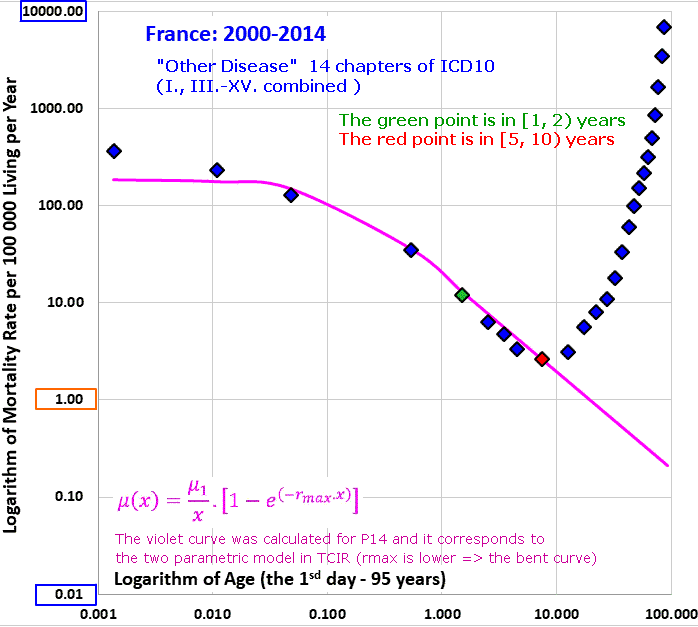

Supplement: Supplementary file 2 [file Data_Sheet_1.ZIP › Animation 5.gif]

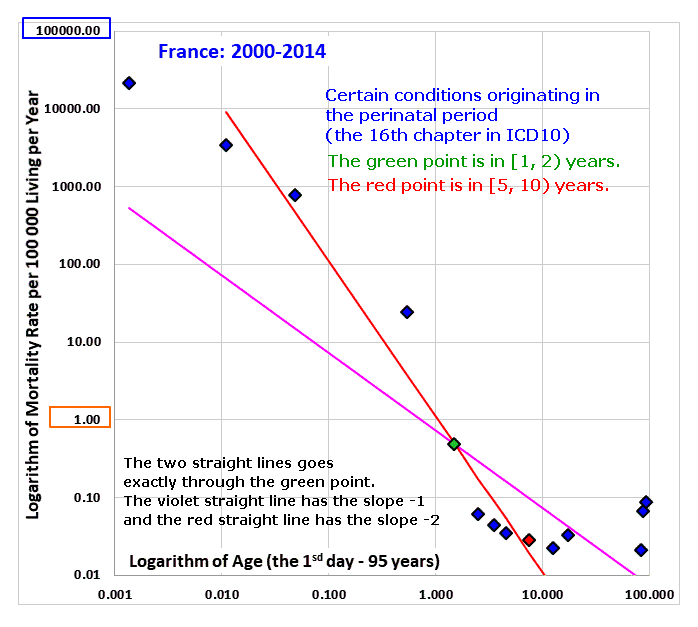

Supplement: Supplementary file 2 [file Data_Sheet_1.ZIP › Animation 6.gif]

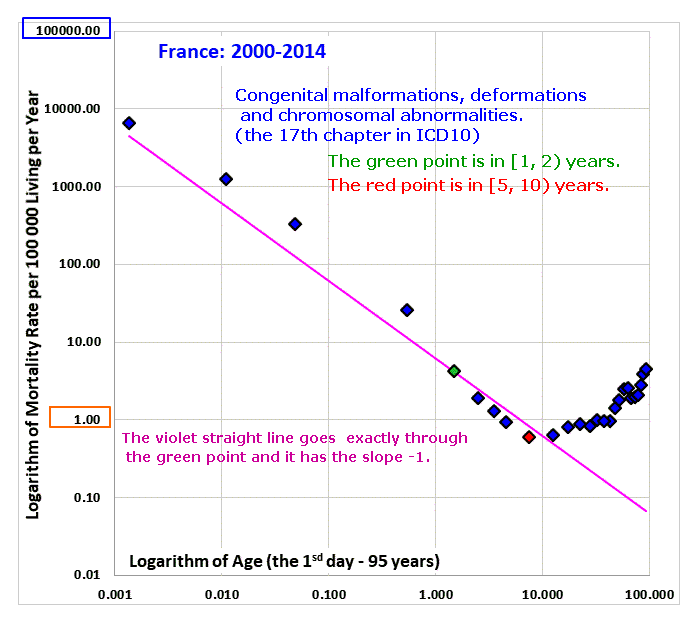

Supplement: Supplementary file 2 [file Data_Sheet_1.ZIP › Animation 7.gif]

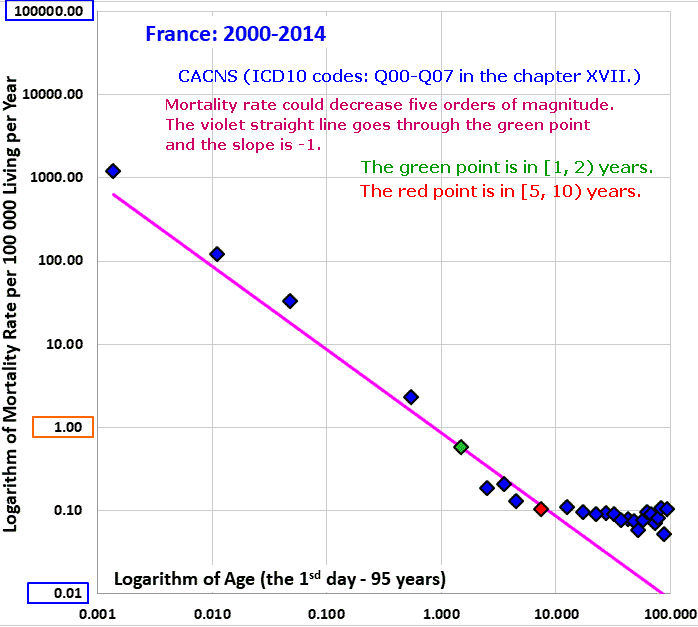

Supplement: Supplementary file 2 [file Data_Sheet_1.ZIP › Animation 8.gif]
