# Supplementary material for: Why Does Child Mortality Decrease With Age? Modeling the Age-Associated Decrease in Mortality Rate Using WHO Metadata From 14 European Countries
Source: Front Pediatr. 2020 Oct 27;8:527811. doi: 10.3389/fped.2020.527811 (PMC7653179; doi:10.3389/fped.2020.527811)
Supplement: Supplementary file 3 [file Data_Sheet_2.docx]

**Appendix**

**Equations in TCIR**

A subpopulation is characterized by the congenital individual risk of death r and by the initial number of born children L_o_(r). The subpopulation is reducing during age x and number of living at age x is:

${L\left( r,x \right)=L}_{o}\left( r \right).exp(-r.x)$ (10)

If continuous formalism is used, the number of all living children at age x is the integral of r values:

$L\left( x \right)=\int_{0}^{\propto} L\left( r,x \right).dr=\int_{0}^{\propto} L_{o}\left( r \right).\exp\left( -r.x \right).dr$ (11)

Mortality rate in the whole population using the definition (1) is:

$\mu\left( x \right)=-\frac{\frac{\partial L\left( x \right)}{\partial x}}{L\left( x \right)}=-\frac{\int_{0}^{\propto} -r.L_{o}\left( r \right).\exp\left( -r.x \right).dr}{\int_{0}^{\propto} L_{o}\left( r \right).\exp\left( -r.x \right).dr}$ (12)

Since the empirical changes of L(x) are very small, if compared to the empirical changes of D(x), the denominator may be replaced by the number of all born children Lo. Empirically, L(x) varies less than 2%, while D(x) varies in the magnitude of more than three orders during the first 20 years. Consequently, the following is valid:

$\mu\left( x \right)\cong\frac{\int_{0}^{\propto} r.L_{o}\left( r \right).\exp\left( -r.x \right).dr}{L_{o}}=\int_{0}^{\propto} r.f\left( r \right).\exp\left( -r.x \right).dr$ (13),

where f(r) is the density function of r at the moment of birth and it is defined by the following formula:

$f\left( r \right)=\frac{L_{o}\left( r \right)}{L_{o}}=\frac{L_{o}\left( r \right)}{\int_{0}^{\propto} L_{o}\left( r \right).dr}$ (14)

The denominator in the formula (14) corresponds to the number of all born people Lo. Mathematically, mortality rate in the whole population at age x is the Laplace transformation of the product r.f(r) in the equation (13). For example, if f(r) is the density function of the log-normal distribution or if the rule “the more severe the impairment, the less frequently it occurs in the born population” (or by the relationship f (r) ≅ constant/r) is valid, the following relationship is:**^24,25^**

$\mu\left( x \right)\cong\int_{0}^{\propto} c.\exp\left( -r.x \right).dr=c.\left[ \frac{\exp\left( -r.x \right)}{-x} \right]_{0}^{\infty}=\frac{c}{x}$ (15)

It has also been shown that, if f(r) is the density function of normal distribution with big variation, or if f(r) is approximately constant in an important interval, mortality rate is given by:**^24,25^**

$\mu\left( x \right)\cong\int_{0}^{\propto} r.c.\exp\left( -r.x \right).dr=c.\left[ \frac{-r.\exp\left( -r.x \right)}{-x}-\frac{exp(-r.x}{\left( -x \right)^{2}} \right]_{0}^{\infty}=\frac{c}{x^{2}}$ (16)

What does "an important interval of r" mean? The theoretical range of r is from zero to infinite, but it is clear that, empirically, it should range between limited values (r_min_, r_max_). For example, if r_max_ is about 1000 and r_min_ is about 0.0001, and the empirical mortality rates is in the population within the age range [0, 20) years is between these values, both formula (15) and (16) are numerically valid (it follows also from simple numerical calculations). In other words, the equations (1), (14) and (15) are weakly affected by the majority of the population with very low r. Consequently, the assumptions about distribution of the r value are, in fact, related only to a small part of the born population with higher r values. For example, the assumption "the more severe the impairment, the less frequently it occurs in the born population" is, in fact, related to the subpopulation with higher r values (this is not true for ATM from CACNS because the age range may be wider and relatively smaller values of r may be important).**^27^** In a simplified way, lower ages correspond to higher values of r while higher ages correspond to lower values of r in the formalisms.

**Other models of ATM after birth in literature**

The first historical model of ATM after birth was formulated by Thiel in 1871.**^36^** He proposed simple exponential decrease of mortality with age. It is concave in the log-log scale and the model was the first term in the following general formula describing all ages:

$\mu\left( x \right)=A.e^{-B.x}\text{ + }{C.e}^{{D.\left( x-E \right)}^{2}}+F.e^{G.x}$ (17)

The formula had tried to describe age trajectory of total mortality from birth up to high ages and the first element is labeled here as "Exp".

Other group of studies had tried to describe the mortality changes with age in the narrower age interval [1, 12) months.**^37-40^** The studies used the following formula for cumulative deaths q(n) up n months:

$q\left( n \right)=a+b.\left[ \ln\left( n+1 \right) \right]^{3}$ for 1 month ≤n ≤ 12 months (18)

The model (18) may be formulated also for mortality rate μ(x) at age x using the definition (1):

$S\left( x \right)=1-\frac{q\left( x \right)}{Lo}=1-\frac{a+b.\left[ \ln\left( 12.x+1 \right) \right]^{3}}{Lo}$ (19)

$\mu\left( x \right)=-\frac{\frac{dS\left( x \right)}{dx}}{S\left( x \right)}=-\frac{\frac{d\left\{ 1-\frac{a+b.\left[ \ln\left( 12.x+1 \right) \right]^{3}}{Lo} \right\}}{dx}}{\left\{ 1-\frac{a+b.\left[ \ln\left( 12.x+1 \right) \right]^{3}}{Lo} \right\}}$ (20)

$\mu\left( x \right)=\frac{\left\{ \left( \frac{b}{Lo} \right).\frac{d\left[ \ln\left( 12.x+1 \right) \right]^{3}}{dx} \right\}}{\left\{ 1-\frac{a+b.\left[ \ln\left( 12.x+1 \right) \right]^{3}}{Lo} \right\}}$ (21)

The model (21) is labeled here as "BP". Additionally, Heligman and Pollard proposed a general relationship for the age all‑causes mortality in 1980, and the following term was suggested for the decline after birth:**^3,7^**

$\mu\left( x \right)=A^{\left( x+B \right)^{C}}\text{ }\text{, for}\text{: 0 < A < 1, 0 < B < 1, 0 < C < 1}$ (22)

The model (22) is also concave in the log-log scale and it is labeled here as "HP".

Besides the three models, the Weibull model (WM) with two parameters is linear in the log-log scale, and may be used to describe mortality decrease with age after birth. WM has assumption that the absolute value of slope has to be less than 1. It is valid for the distribution function F(x) of ages of died people in WM:

$F\left( x \right)=1-S\left( x \right)=1-e^{\left( \frac{-x^{m}}{a} \right)}$ for a > 0 and m > 0 (23)

Furthermore, mortality rate μ(x) at age x using the definition (1) is:

$\mu\left( x \right)=-\frac{\frac{dS\left( x \right)}{dx}}{S\left( x \right)}=-m.\left( \frac{-x^{m}}{a} \right).\frac{e^{\left( \frac{-x^{m}}{a} \right)}}{e^{\left( \frac{-x^{m}}{a} \right)}}=-m.\left( \frac{-x^{m}}{a} \right)=\mu_{1}x^{m-1}$ (24)

Slope equals to m -1 in the log-log scale and parameter m is zero if the inverse proportion is valid. On the other hand, if m is equal or less than 0 (if slope is equal or less than -1) then F(x) does not increase with age x and it is not distribution function. Consequently, WM may not be used if m=1 or m< -1, if m > 1 then the linear model (3) corresponds to the Weibull distribution.

**Table 4 P-values of two tests calculated in the chapter Neoplasms (II.).**

| **Population** | **c/x in the first year** | **Age-independence in (1, 10] years** |
| --- | --- | --- |
| France | 0.14 | 0.49 |
| Germany | 0.11 | 0.39 |
| Italy | 0.12 | 0.13 |
| Spain | 0.01 | 0.33 |
| UK | 0.04 | 0.76 |
| **P1** | 0.07 | 0.46 |
| Czech Republic | 0.04 | 0.22 |
| Austria | 0.43 | 0.97 |
| Hungary | 0.17 | 0.66 |
| Poland | 0.07 | 0.26 |
| Slovakia | 0.03 | 0.31 |
| **P2** | 0.08 | 0.11 |
| Sweden | 0.20 | 0.81 |
| Norway | 0.12 | 0.48 |
| Denmark | 0.19 | 0.21 |
| Finland | 0.62 | 0.97 |
| **P3** | 0.26 | 0.43 |
| **P14** | 0.08 | 0.99 |

**Notes**: *The column labeled as "****c/x in the first year****" contains p-values of the test of the null hypothesis Ho: γ= -1 in the log-log scale in the age interval [0, 365) days. The last column contains p-values of the test of the null hypothesis Ho: γ=0 in the log-log scale in the age interval [1, 10) years.*

**Table 5 Results calculated in the chapter "Certain conditions originating in the perinatal period" (XVI.) calculated in the age interval [0, 15) years in the log-log scale**

| **Population** | **Test of linearity** | **γ** | **Lower CI 95%** | **Upper CI 95%** | **^2^** | **Test of c/(x.x)** |
| --- | --- | --- | --- | --- | --- | --- |
| France | 0.109 | -1.728 | -2.034 | -1.423 | 0.9495 | 0.074 |
| Germany | 0.203 | -1.576 | -1.788 | -1.364 | 0.9702 | 0.002 |
| Italy | 0.175 | -1.586 | -1.807 | -1.364 | 0.9679 | 0.003 |
| Spain | 0.211 | -1.558 | -1.810 | -1.307 | 0.9575 | 0.004 |
| UK | 0.026 | x | x | x | x | x |
| **P1** | 0.116 | -1.615 | -1.841 | -1.390 | 0.9680 | 0.004 |
| Czech Republic |  |  |  |  |  |  |
| Austria | 0.709 | -1.500 | -1.715 | -1.286 | 0.9664 | 0.001 |
| Hungary |  |  |  |  |  |  |
| Poland | 0.005 | x | x | x | x | x |
| Slovakia |  |  |  |  |  |  |
| **P2** | 0.069 | -1.802 | -2.132 | -1.472 | 0.9460 | 0.204 |
| Sweden | 0.217 | -1.423 | -1.581 | -1.265 | 0.9794 | 0.000 |
| Norway | 0.166 | -1.470 | -1.643 | -1.298 | 0.9771 | 0.000 |
| Denmark | 0.531 | -1.460 | -1.602 | -1.319 | 0.9843 | 0.000 |
| Finland |  |  |  |  |  |  |
| **P3** | 0.150 | -1.467 | -1.605 | -1.330 | 0.9852 | 0.000 |
| **P14** | 0.100 | -1.629 | -1.862 | -1.396 | 0.9663 | 0.006 |

**Notes**: *The second column labeled as "****Test of linearity****" contains p-values of the test of the null hypothesis for the quadratic element H_o_: δ = 0 in model (2) (all ATM except in the U.K. and Poland were linear in the log-log scale). The column labeled as "****γ****" contains point estimations of the slopes in model (3) and the next two columns contain limits of 95% confidence interval of the parameter* ***γ****.* ***^2^*** *is adjusted coefficient of determination calculated for two parameters and nine points in model (3). The last column labeled as "****Test of c/(x.x)****" contains p-values of the standard Fisher’s test that the model (3) with two parameters does not provide a significantly better fit than the same model with the slope -2 in the log-log scale (the model with the slope -2 was valid in France and P2).*

**Table 6 Results calculated in the chapter "Congenital malformations, deformations and chromosomal abnormalities" (XVII.) in the age interval [0, 15) years in the log-log scale**

| **Population** | **Test of linearity** | **γ** | **Lower CI 95%** | **Upper CI 95%** | **^2^** | **R_b_^2^** | **R_b_^2^-^2^** | **Test of c/x** |
| --- | --- | --- | --- | --- | --- | --- | --- | --- |
| France | 0.170 | -1.113 | -1.217 | -1.009 | 0.9854 | 0.9769 | -0.0085 | 0.037 |
| Germany | 0.211 | -1.069 | -1.151 | -0.986 | 0.9901 | 0.9871 | -0.0030 | 0.091 |
| Italy | 0.112 | -1.080 | -1.185 | -0.975 | 0.9842 | 0.9806 | -0.0036 | 0.118 |
| Spain | 0.082 | -1.049 | -1.170 | -0.927 | 0.9777 | 0.9781 | 0.0003 | 0.381 |
| UK | 0.384 | -1.123 | -1.208 | -1.038 | 0.9904 | 0.9796 | -0.0108 | 0.010 |
| **P1** | 0.181 | -1.090 | -1.184 | -0.997 | 0.9876 | 0.9822 | -0.0054 | 0.057 |
| Czech Republic | 0.033 | x | x | x | x | x | x | x |
| Austria | 0.946 | -1.047 | -1.136 | -0.958 | 0.9878 | 0.9872 | -0.0006 | 0.261 |
| Hungary | 0.067 | -1.026 | -1.127 | -0.925 | 0.9839 | 0.9851 | 0.0011 | 0.565 |
| Poland | 0.101 | -1.115 | -1.202 | -1.027 | 0.9897 | 0.9803 | -0.0093 | 0.016 |
| Slovakia | 0.010 | x | x | x | x | x | x | x |
| **P2** | 0.099 | -1.087 | -1.173 | -1.000 | 0.9894 | 0.9842 | -0.0051 | 0.050 |
| Sweden | 0.474 | -1.092 | -1.211 | -0.973 | 0.9803 | 0.9754 | -0.0048 | 0.111 |
| Norway | 0.178 | -1.063 | -1.138 | -0.988 | 0.9916 | 0.9890 | -0.0025 | 0.091 |
| Denmark | 0.048 | x | x | x | x | x | x | x |
| Finland | 0.951 | -1.080 | -1.151 | -1.009 | 0.9927 | 0.9881 | -0.0046 | 0.032 |
| **P3** | 0.323 | -1.076 | -1.152 | -1.000 | 0.9917 | 0.9877 | -0.0040 | 0.050 |
| **P14** | 0.161 | -1.088 | -1.177 | -0.998 | 0.9887 | 0.9835 | -0.0052 | 0.053 |

**Notes***: The column labeled as "****Test of linearity****" contains p-values of the test of the null hypothesis for the quadratic element H_o_: δ = 0 in model (2) (all ATM except in the Czech Republic, Slovakia and Denmark were linear in the log-log scale). The column labeled as "****γ****" contains point estimations of slopes in model (3) and the next two columns contain limits of 95% confidence interval of the parameter* ***γ****.* ***^2^*** *is adjusted coefficient of determination calculated for two parameters and nine points in the model (3).* ***R_b_^2^*** *is coefficient of determination calculated in the inverse proportion (4) in the log-log scale, and it was calculated using formula (6). The last column labeled as "****Test of c/(x)****" contains p-values of the standard Fisher’s test that the model (3) with two parameters does not provide a significantly better fit than the same model with the slope -1 in the log-log scale (the inverse proportion (4) was valid for all ATM except in France, the U.K., Slovakia, Denmark and Finland).*

**Table 7 Results calculated in the group CACNS in the age interval [0, 15) years in the log-log scale**

| **Population** | **Test of linearity** | **γ** | **Lower CI 95%** | **Upper CI 95%** | **^2^** | **R_b_^2^** | **R_b_^2^-^2^** | **Test of c/x** |
| --- | --- | --- | --- | --- | --- | --- | --- | --- |
| France | 0.747 | -1.092 | -1.180 | -1.004 | 0.9891 | 0.9832 | -0.0059 | 0.042 |
| Germany | 0.017 |  |  |  |  |  |  |  |
| Italy | 0.033 |  |  |  |  |  |  |  |
| Spain | 0.648 | -0.937 | -1.020 | -0.854 | 0.9869 | 0.9839 | -0.0029 | 0.121 |
| UK | 0.081 | -1.051 | -1.112 | -0.989 | 0.9943 | 0.9926 | -0.0017 | 0.094 |
| **P1** | 0.127 | -0.998 | -1.062 | -0.934 | 0.9931 | 0.9938 | 0.00077 | 0.949 |
| Czech Republic | 0.136 | -0.883 | -0.951 | -0.815 | 0.9901 | 0.9739 | -0.0162 | 0.004 |
| Austria | 0.014 |  |  |  |  |  |  |  |
| Hungary | 0.371 | -0.827 | -0.911 | -0.744 | 0.9830 | 0.9421 | -0.041 | 0.001 |
| Poland | 0.041 |  |  |  |  |  |  |  |
| Slovakia | 0.030 |  |  |  |  |  |  |  |
| **P2** | 0.120 | -0.943 | -1.013 | -0.872 | 0.9907 | 0.9880 | -0.0027 | 0.096 |
| Sweden | 0.342 | -0.939 | -1.028 | -0.851 | 0.9852 | 0.9827 | -0.0025 | 0.151 |
| Norway | 0.190 | -0.954 | -1.086 | -0.822 | 0.9686 | 0.9699 | 0.00124 | 0.445 |
| Denmark | 0.152 | -0.991 | -1.060 | -0.923 | 0.9920 | 0.9928 | 0.00081 | 0.780 |
| Finland | 0.013 |  |  |  |  |  |  |  |
| **P3** | 0.078 | -0.949 | -1.040 | -0.859 | 0.9848 | 0.9837 | -0.0011 | 0.231 |
| **P14** | 0.065 | -0.975 | -1.035 | -0.915 | 0.9937 | 0.9937 | 0.00004 | 0.360 |

**Notes**: *The column labeled as "****Test of linearity****" contains p-values of the test of the null hypothesis for the quadratic element H_o_: δ = 0 in model (2) (all ATM were linear in the log-log scale except in Germany, Italy, Austria, Poland, Slovakia and Finland). The column labeled as "****γ****" contains point estimations of slopes in model (3) and the next two columns contain limits of 95% confidence interval of the parameter* ***γ****.* ***^2^*** *is adjusted coefficient of determination calculated for two parameters and nine points in the model (3).* ***R_b_^2^*** *is coefficient of determination calculated in the inverse proportion (4) in the log-log scale, and it was calculated using formula (6). The last column labeled as "****Test of c/(x)****" contains p-values of the standard Fisher’s test that the model (3) with two parameters does not provide a significantly better fit than the same model with the slope -1 in the log-log scale (the inverse proportion (4) was valid for all 12 ATM except in France, the Czech republic and Hungary).*

**Table 8 Parameters of bending age trajectory of mortality calculated in the model (15) in P14**

|  | **Chapter** | **ATM** | **r_max_** | **μ_1_** | **^2^** |
| --- | --- | --- | --- | --- | --- |
| I. | Certain infectious and parasitic diseases | yes | 6.1 | 2.94 | 0.977 |
| II. | Neoplasms | yes | x | x | x |
| III. | Diseases of the blood and blood-forming organs and certain disorders involving the immune mechanism | yes | 12.8 | 0.95 | 0.908 |
| IV. | Endocrine, nutritional and metabolic diseases | yes | 20.6 | 2.47 | 0.975 |
| V. | Mental and behavioural disorders | no | x |  | x |
| VI. | Diseases of the nervous system | yes | 5.1 | 5.88 | 0.966 |
| VII. | Diseases of the eye and adnexa | no | x | x | x |
| VIII. | Diseases of the ear and mastoid process | no | x | x | x |
| IX. | Diseases of the circulatory system | yes | 17.2 | 2.50 | 0.938 |
| X. | Diseases of the respiratory system | yes | 4.5 | 3.29 | 0.917 |
| XI. | Diseases of the digestive system | yes | 14.5 | 1.00 | 0.939 |
| XII. | Diseases of the skin and subcutaneous tissue | no | x | x | x |
| XIII. | Diseases of the musculoskeletal system and connective tissue | no | x | x | x |
| XIV. | Diseases of the genitourinary system | yes | 17.0 | 0.23 | 0.963 |
| XV. | Pregnancy, childbirth and the puerperium | no | x | x | x |
| XVI. | Certain conditions originating in the perinatal period | yes | x | x | x |
| XVII. | Congenital malformations, deformations and chromosomal abnormalities | yes | x | x | x |
| XVIII. | Symptoms, signs and abnormal clinical and laboratory findings, not elsewhere classified | yes | 563.1 | 3.19 | 0.935 |
| XIX.-XXI. | Accidents | yes | x | x | x |
|  | "Other diseases" | yes | 9.6 | 19.61 | 0.968 |

**Notes**: *The category labeled as "Other diseases" contains the chapters: I.,III.-XV. (without Neoplasms (II.)). The column labeled as "****ATM****" indicates if ATM was obtained. The columns labeled as "****r_max_****" and "****μ_1_****" contain point estimations of the two parameters calculated using model (8).* ***^2^*** *is adjusted coefficient of determination calculated for two parameters and nine points in the model (8).*

**Table 9. Proportions of subcategories of CACNS in age categories**

| **Age** | **Q00** | **Q01** | **Q02** | **Q03** | **Q04** | **Q05** | **Q06** | **Q07** |
| --- | --- | --- | --- | --- | --- | --- | --- | --- |
| 1d | 42.7 | 6.7 | 1.9 | 13.9 | 18.6 | 13.1 | 0.1 | 2.9 |
| 1-7d | 23.2 | 6.7 | 3.0 | 17.6 | 32.7 | 13.8 | 0.5 | 2.5 |
| 7-28d | 5.6 | 4.2 | 3.1 | 24.4 | 45.6 | 11.1 | 1.4 | 4.6 |
| 28-365d | 2.0 | 2.6 | 6.6 | 25.1 | 48.8 | 6.3 | 1.8 | 6.8 |
| 1 -2 years | 1.4 | 2.0 | 11.9 | 27.0 | 44.9 | 5.2 | 0.3 | 7.4 |
| 2 -3 years | 0.9 | 1.8 | 12.6 | 30.3 | 43.0 | 5.4 | 0.4 | 5.6 |
| 3 - 4 years | 0.6 | 1.6 | 14.2 | 26.5 | 43.8 | 4.7 | 0.6 | 7.9 |
| 4 -5 years | 0.8 | 1.6 | 11.5 | 29.9 | 43.4 | 6.6 | 0.0 | 6.1 |
| 5 -10 years | 0.9 | 2.0 | 13.3 | 28.0 | 43.6 | 4.8 | 0.7 | 6.7 |
| 10 -15 years | 0.6 | 2.5 | 13.9 | 25.0 | 41.4 | 7.6 | 0.8 | 8.1 |
| 15-20 years | 0.6 | 1.5 | 15.2 | 22.3 | 38.0 | 13.4 | 0.4 | 8.7 |
| 20 -25 years | 0.2 | 1.4 | 14.3 | 20.9 | 33.9 | 19.2 | 0.9 | 9.3 |
| 25 -30 years | 0.4 | 1.3 | 13.0 | 22.5 | 28.4 | 26.1 | 0.4 | 7.9 |
| 30 -35 years | 0.4 | 1.1 | 12.2 | 14.5 | 28.4 | 34.3 | 0.8 | 8.3 |
| 35 -40 years | 0.0 | 0.9 | 9.3 | 18.2 | 27.1 | 38.0 | 0.5 | 6.1 |
| 40 - 45 years | 0.0 | 0.8 | 4.7 | 15.9 | 29.9 | 39.6 | 0.4 | 8.7 |
| 45 - 50 years | 0.2 | 1.1 | 7.0 | 17.5 | 29.8 | 33.3 | 1.1 | 10.1 |
| 50 -55 years | 0.3 | 1.6 | 5.2 | 18.8 | 27.7 | 29.5 | 0.3 | 16.7 |
| 55 -60 years | 0.0 | 0.3 | 3.7 | 23.5 | 35.8 | 22.5 | 0.9 | 13.3 |
| 60 - 65 years | 0.3 | 0.7 | 4.6 | 29.0 | 33.7 | 13.5 | 2.0 | 16.2 |
| 65 -70 years | 0.0 | 1.1 | 5.1 | 24.9 | 32.1 | 16.6 | 1.4 | 18.8 |
| 70 -75 years | 1.3 | 0.9 | 3.0 | 21.0 | 35.2 | 17.6 | 3.4 | 17.6 |
| 75 -80 years | 0.0 | 3.8 | 1.1 | 26.1 | 33.7 | 10.9 | 1.6 | 22.8 |
| 80 - 85 years | 0.0 | 3.4 | 2.5 | 18.5 | 38.7 | 15.1 | 5.0 | 16.8 |

**Notes**: *100% in each row represent all deaths from all subcategories of CACNS in specific age category*. *The subcategories are: Q00="Anencephaly and similar malformations"; Q01="Encephalocele"; Q02="Microcephaly"; Q03="Congenital hydrocephalus"; Q04="Other congenital malformations of the brain"; Q05="Spina bifida"; Q06="Other congenital malformations of spinal cord"; Q07="Other congenital malformations of nervous system".*
